# Identification of CD98 as a Novel Biomarker for HIV-1 Permissiveness and Latent Infection

**DOI:** 10.1128/mbio.02496-22

**Published:** 2022-10-10

**Authors:** Wanying Zhang, Mo Zhou, Cancan Chen, Shiyu Wu, Lilin Wang, Baijin Xia, Jun Liu, Xiancai Ma, Ting Pan, Hui Zhang, Linghua Li, Bingfeng Liu

**Affiliations:** a Institute of Human Virology, Key Laboratory of Tropical Disease Control of Ministry of Education, Guangdong Engineering Research Center for Antimicrobial Agent and Immunotechnology, Zhongshan School of Medicine, Sun Yat-sen University, Guangzhou, Guangdong, China; b Infectious Diseases Center, Guangzhou Eighth People’s Hospital, Guangzhou Medical University, Guangzhou, China; c Department of Pathology, The First Affiliated Hospital, Sun Yat-sen University, Guangzhou, China; d Shenzhen Blood Center, Shenzhen, Guangdong, China; e Qianyang Biomedical Research Institute, Guangzhou, Guangdong, China; Columbia University Medical College

**Keywords:** CD98, latent reservoir, human immunodeficiency virus, HIV-1 infection, cellular biomarker

## Abstract

Human immunodeficiency virus type 1 (HIV-1) can integrate viral DNA into host cell chromosomes to establish a long-term stable latent reservoir, which is a major obstacle to cure HIV-1 infection. The characteristics of the HIV-1 latent reservoir have not been fully understood. Here, we identified 126 upregulated plasma membrane proteins in HIV-1 latently infected cells by a label-free liquid chromatography-tandem mass spectrometry analysis. The higher levels of CD98 expression in multiple HIV-1 latently infected cell lines and primary CD4^+^ T cells compared to uninfected cells were further confirmed by quantitative reverse transcription PCR (RT-qPCR) and flow cytometry analyses. In addition, CD98^high^ CD4^+^ T cells displayed hyper-permissiveness to HIV-1 infection and possessed distinct immune phenotypic profiles associated with Th17 and peripheral follicular T helper (pTFH) characteristics. Notably, the CD98^high^ resting memory CD4^+^ T cells harbored significantly higher cell-associated viral RNA and intact provirus than CD98^low^ counterparts in HIV-1-infected individuals receiving combined antiretroviral therapy. Furthermore, CD98^high^ CD4^+^ T cells exhibited a robust proliferative capacity and significantly contributed to the clonal expansion of the HIV-1 latent reservoir. Our study demonstrates that CD98 can be used as a novel biomarker of HIV-1 latently infected cells to indicate the effect of various strategies to reduce the viral reservoir.

## INTRODUCTION

Human immunodeficiency virus type 1 (HIV-1) is a critical pathogen that causes AIDS. In 2020, 1.5 million new HIV-1 infections and 680,000 deaths from HIV-1 related complications were reported globally (https://www.who.int/data) (1). The combination antiretroviral therapy (cART) significantly suppresses HIV-1 to an undetectable level in the blood, improves immune function, delays the progression of disease, and decreases the mortality in HIV-1-infected individuals (2, 3). However, owing to the persistence of the HIV-1 latent reservoir, viremia rebounds rapidly within a few weeks after cART is interrupted. Therefore, the eradication of the viral latent reservoir is a major obstacle in curing HIV-1 infection (2). The persistence of the HIV-1 latent reservoir is due to a small proportion of CD4^+^ T cells harboring the integrated HIV-1 viral genome. These cells range from 0.1 to 10 in every million CD4^+^ T cells. They can persist for the long-term within HIV-1-infected individuals receiving cART and thus constitute the HIV-1 reservoir. They are quite stable, and the approximate half-life is 44 months (2, 4). As these cells do not express any viral proteins on the cellular membrane, the identification of relatively specific host protein(s) is important, as it could guide the development of robust and efficient eradication strategies (5). Although many researchers have made great efforts to discover characteristic biomarkers of the HIV-1 reservoir in the past 2 decades, this remains a challenging task (6–8).

Several membrane-bound molecules, such as CD32a, CD30, CD2, PD-1, LAG-3, and TIGIT, were reported as the HIV-1 latency biomarkers (9–11). However, these biomarkers are not specifically enriched in HIV-1 latently infected cells; therefore, more cell plasma membrane proteins related to the characteristics of the HIV-1 latent reservoir need to be further elucidated. In this study, we identified higher levels of CD98 expression on HIV-1 latently infected cells using label-free liquid chromatography-tandem mass spectrometry (LC-MS/MS) analyses. We then determined that CD98^high^ CD4^+^ T cells were highly permissive for HIV-1 infection compared to CD98^low^ CD4^+^ T cells. Moreover, CD4^+^ T cells with high CD98 expression displayed distinct immune phenotypes. More importantly, the CD98^high^ resting memory CD4^+^ T cells harbored more HIV-1 proviruses and possessed a significantly higher clonal expansion capacity than CD98^low^ counterparts from HIV-1-infected individuals receiving cART. Overall, our study demonstrates that the high expression of CD98 is a novel characteristic of the HIV-1 latent reservoir.

## RESULTS

### Identification of plasma membrane proteins differentially expressed on HIV-1 latently infected cells compared to uninfected cells.

**FIG 1:**
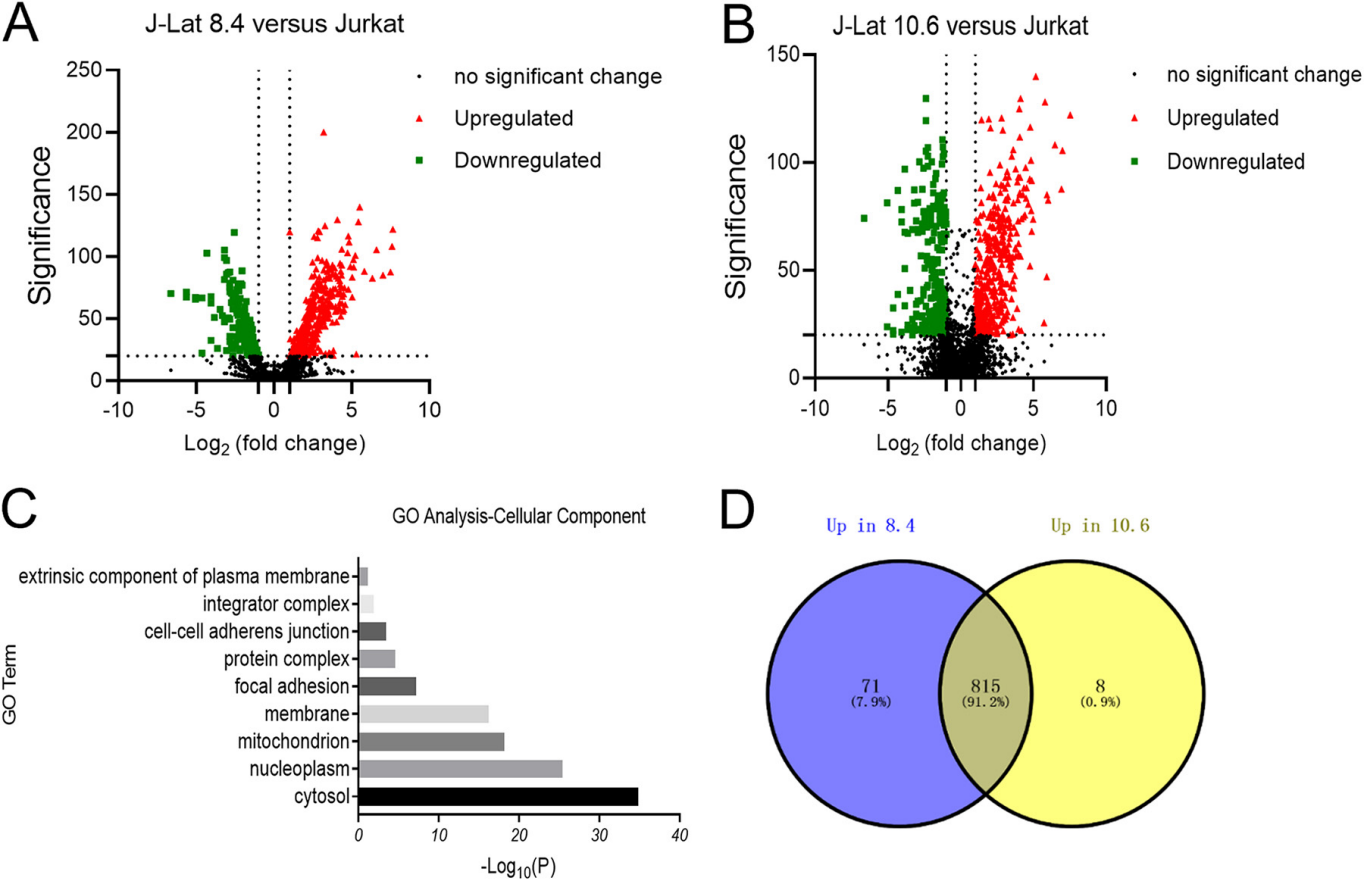
Proteomic profile analyses of HIV-1 latently infected cells. (A and B) Volcano plots of LC-MS/MS analysis of HIV-1 latently infected J-Lat 8.4 cells (A) and J-Lat 10.6 cells (B); upregulated proteins are denoted by red triangles, downregulated proteins are denoted by green squares, and proteins with no significant change are denoted by black dots. Dashed lines on the *x* axis indicate log_2_ (fold change) of 1 or −1; a dashed line on the *y* axis indicates significance of 20. (C) Gene ontology analysis term “cellular component” enriched among proteins in upregulated proteins in HIV-1 latently infected cells. (D) Venn diagram of the upregulated proteins in HIV-1 latently infected cells compared to uninfected cells.

J-Lat cell lines (J-Lat 8.4 or 10.6), which contain a near-full-length integrated HIV-1 genome, are the most popular HIV-1 latently infected cell line models (12). To identify differential expression proteins between HIV-1 latently infected J-Lat cells and uninfected Jurkat T cells, we performed whole-cell lysed LC-MS/MS analyses. Compared to Jurkat T cells, 1,291 and 1,224 differentially expressed proteins were identified in J-Lat 8.4 and in J-Lat 10.6, respectively (Fig. 1A and B). Venn diagrams showed that 815 proteins were upregulated (Fig. 1D), and 255 proteins were downregulated in both HIV-1 latently infected cell lines compared to Jurkat T cells (significance, ≥20, fold change, ≥2).

We determined the gene ontology “cellular component,” “biological process,” and “molecular function” term annotations of the 815 upregulated proteins in both HIV-1 latently infected cell lines using the Database for Annotation, Visualization, and Integrated Discovery (Fig. 1C; Fig. S1A and B` in the supplemental material). Candidate proteins were exhibited in volcano plots and Venn diagrams between HIV-1 latently infected cells and uninfected cells (Fig. 1A, B and D). In the gene ontology analysis, we noted that some upregulated proteins were related to membrane, cell-cell adhesion, and protein binding. Further analysis showed that 126 plasma membrane proteins were upregulated in HIV-1 latently infected cells. The complete proteomic profiling analysis of upregulated plasma membrane proteins on HIV latently infected cells is shown in Table S1 in the supplemental material.

10.1128/mbio.02496-22.1 FIG S1 Upregulated expression proteins in HIV latently infected cells compared to uninfected cells. (A and B) Gene ontology analyses of upregulated expression proteins in J-Lat 8.4 and J-Lat 10.6 cells compared to Jurkat T cells, including the terms “biology process” (A) and “molecular function” (B). (C and D) The expression levels of CD81 and CD71 were measured by flow cytometry. The representative histograms are shown. MFI, median fluorescence intensity. Data represent the means ± SD from at least triplicates. *P* values were calculated by Student’s *t* test. ***, *P < *0.001; ****, *P < *0.0001. Download FIG S1, TIF file, 0.8 MB. Copyright © 2022 Zhang et al. 2022 Zhang et al. https://creativecommons.org/licenses/by/4.0/ This content is distributed under the terms of the Creative Commons Attribution 4.0 International license.

10.1128/mbio.02496-22.7 TABLE S1 Upregulated plasma membrane proteins on HIV latently infected cells. Download Table S1, DOCX file, 0.03 MB. Copyright © 2022 Zhang et al. 2022 Zhang et al. https://creativecommons.org/licenses/by/4.0/ This content is distributed under the terms of the Creative Commons Attribution 4.0 International license.

Considering both the fold change of expression and the available commercial flow cytometric antibodies to detect these proteins, seven plasma membrane proteins, BST2, CD123, CD1b, CD36, CD81, CD98, and TFR1, were selected among those with an upregulated expression in HIV-1 latently infected cells compared to that in uninfected cells as determined by LC-MS/MS analyses (Table 1). Some of these proteins are related to HIV-1 infection. For example, BST2, also named CD317, has been reported as a viral restriction factor that can inhibit HIV-1 virion release from the cell surface and can be used as a cellular biomarker for HIV-1 permissiveness (13, 14). In addition, CD81 belongs to a member of the tetraspanin family that can block HIV-1-induced syncytia formation and viral entry and enhances HIV-1 reverse transcription (15). However, there are few reports on other proteins involved in HIV-1 infection.

**TABLE 1:** Candidate plasma membrane proteins upregulated in HIV-1 latently infected cells

UniProtKB accession no.	Significance	Group profile (ratio)^*a*^	Description
O75794	57.08	11.08:7.58:1.00	Cell division cycle protein 123 homolog OS = Homo sapiens GN = CDC123 PE = 1 SV = 1
E9PJK1	52.08	4.59:4.96:1.00	CD81 antigen OS = Homo sapiens GN = CD81 PE = 1 SV = 1
P08195	35.53	2.73:2.77:1.00	Isoform 4 of 4F2 cell-surface antigen heavy chain OS = Homo sapiens GN = SLC3A2
H7C0I2	21.82	4.34:5.63:1.00	T-cell surface glycoprotein CD1b (fragment) OS = Homo sapiens GN = CD1B PE = 4 SV = 1
Q10589	6.84	2.65:1.01:1.00	Bone marrow stromal antigen 2 OS = Homo sapiens GN = BST2 PE = 1 SV = 1
Q14108	2.84	1.61:1.90:1.00	Lysosome membrane protein 2 OS = Homo sapiens GN = SCARB2 PE = 1 SV = 2
G3V0E5	2.37	1.25:1.42:1.00	Transferrin receptor (P90 CD71) isoform CRA_c OS = Homo sapiens GN = TFRC PE = 1 SV = 1

a Group profile (ratio): J-Lat 10.6: J-Lat 8.4: Jurkat T cells.

### The expression of CD98 is upregulated on multiple HIV-1 latently infected cells.

**FIG 2:**
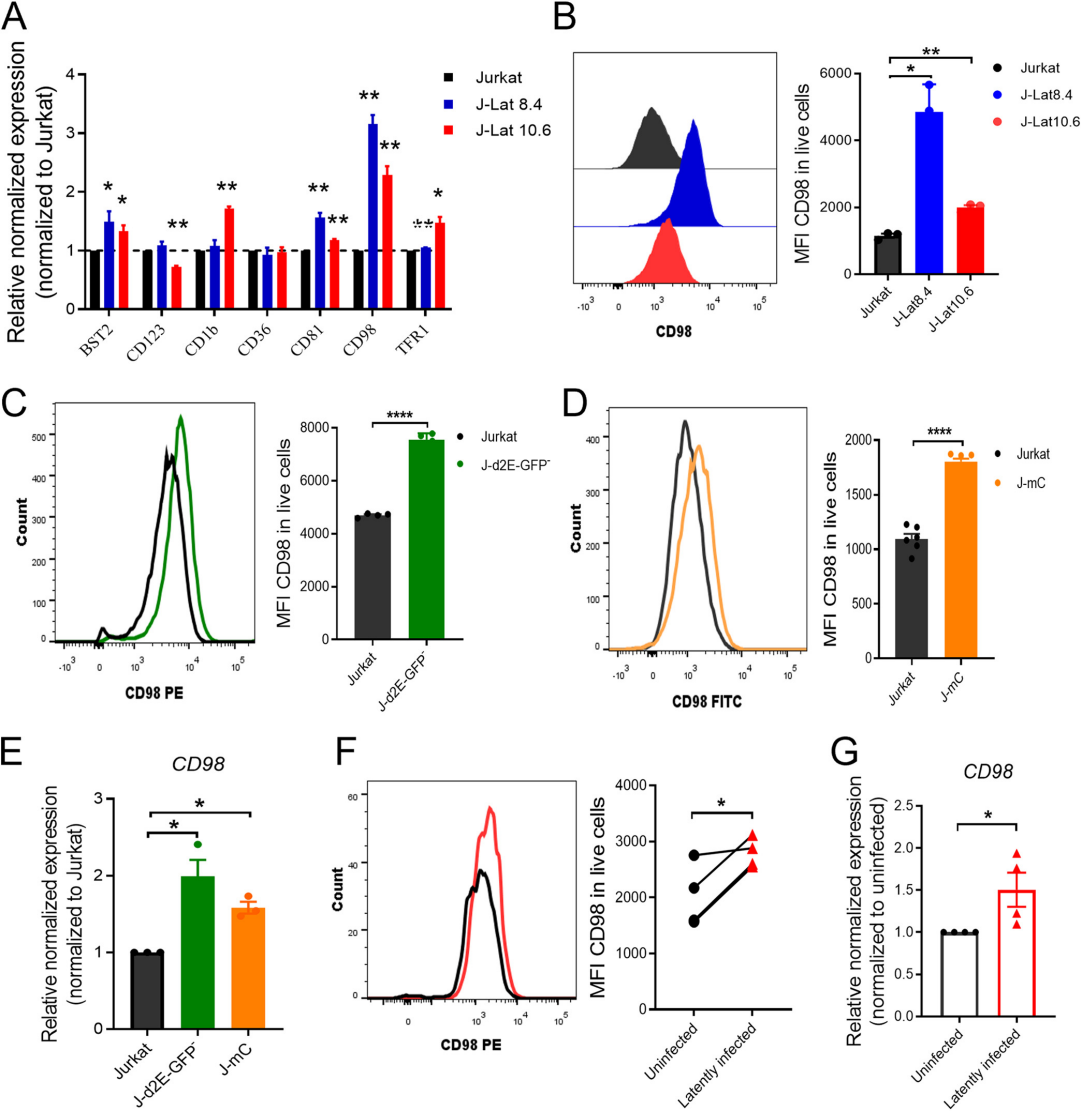
The expression of CD98 on multiple HIV-1 latency models was upregulated. (A) The expression of seven candidate plasma membrane proteins on HIV-1 latently infected cells and uninfected cells. (B to D) Representative histograms showing CD98 expression on HIV latently infected cells, including J-Lat cell lines (B), J-d2E-GFP- cells (C), and J-mC cells (D), compared to Jurkat T cells. (E) The levels of mRNA expression of *CD98* on HIV-1 latently infected cells compared to uninfected cells, including J-d2E-GFP- cells and J-mC cells. (F and G) The expression of CD98 on HIV-1 latently infected primary CD4^+^ T cells compared to uninfected cells at protein level (F) and mRNA level (G) (*n* = 4). The levels of mRNA expression normalized to that for *β-actin* were measured by RT-qPCR. The representative plots are shown. MFI, mean fluorescence intensity. Data represent means ± SD from at least triplicates. *P* values were calculated by Student's *t* test or Mann-Whitney U test. *, *P < *0.05; **, *P < *0.01; ***, *P < *0.001; ****, *P < *0.001.

To validate the LC/MS-MS results, we performed RT-qPCR and flow cytometry experiments. We confirmed that CD81 and transferrin receptor (TFR1; also known as CD71) were upregulated in HIV-1 latently infected cells (Fig. 2A and Fig. S1C and D). The expression of CD98 was the most significantly upregulated in HIV-1 latently infected cells (J-Lat 8.4 and 10.6) among these plasma membrane proteins (Fig. 2A). CD98 (CD98hc, also known as SLC3A2, 4F2) is a type II transmembrane glycoprotein with multiple functions, including integrin signaling and amino acid transport. However, the role of CD98 in HIV-1 latent infection remains unclear.

Considering that J-Lat cell lines are single cell clones with one known HIV-1 genome integration site, we further detected the expression of CD98 in two other types of HIV-1 latency cell models with multiple HIV-1 genome integration sites: J-d2E-GFP^–^ cells, which were developed by us (Fig. S2), and J-mC cells (16). Flow cytometry and RT-qPCR analyses showed that the expression levels of CD98 were significantly higher in HIV-1 latently infected cell lines than in uninfected cells (Fig. 2B to E).

10.1128/mbio.02496-22.2 FIG S2 Strategy of establishing an HIV-1 latently infected cell line with multiple HIV-1 integration sites. (A) Diagram of the d2E-GFP genome based on the original HIV-1_NL4-3_ strain. (B) Experimental procedures of establishing an HIV latency model called J-d2E-GFP^–^ based on the Jurkat T cell line. (C) Reactivation of J-d2E-GFP^–^ cells with different stimuli for 24 h. The proportions of the GFP-positive population indicating reactivation efficiency are shown in the bottom right corners. Download FIG S2, TIF file, 1.1 MB. Copyright © 2022 Zhang et al. 2022 Zhang et al. https://creativecommons.org/licenses/by/4.0/ This content is distributed under the terms of the Creative Commons Attribution 4.0 International license.

To further confirm our observations for CD98, we used primary CD4^+^ T cells from peripheral blood mononuclear cells (PBMCs) of healthy donors to establish HIV-1 latency models according to a previously described method (10). Briefly, we infected primary CD4^+^ T cells with HIV-1_NL4-3-GFP_ virus after T cell receptor (TCR) stimulation for 48 h, followed by culture for 1 week in a medium containing interleukin-2 (IL-2). Subsequently, they were allowed to quiesce for another week in the presence of a low concentration of IL-7. The expression of GFP or CD98 was detected by flow cytometry at the indicated time points. The results were in line with the T cell line results, and the expression of CD98 in HIV-1 latently infected primary CD4^+^ T cells was significantly higher than that in uninfected cells at both the protein and mRNA levels (Fig. 2F and G).

### Differential transcriptomic profile analysis between CD98^high^ CD4^+^ T cells and CD98^low^ CD4^+^ T cells.

**FIG 3:**
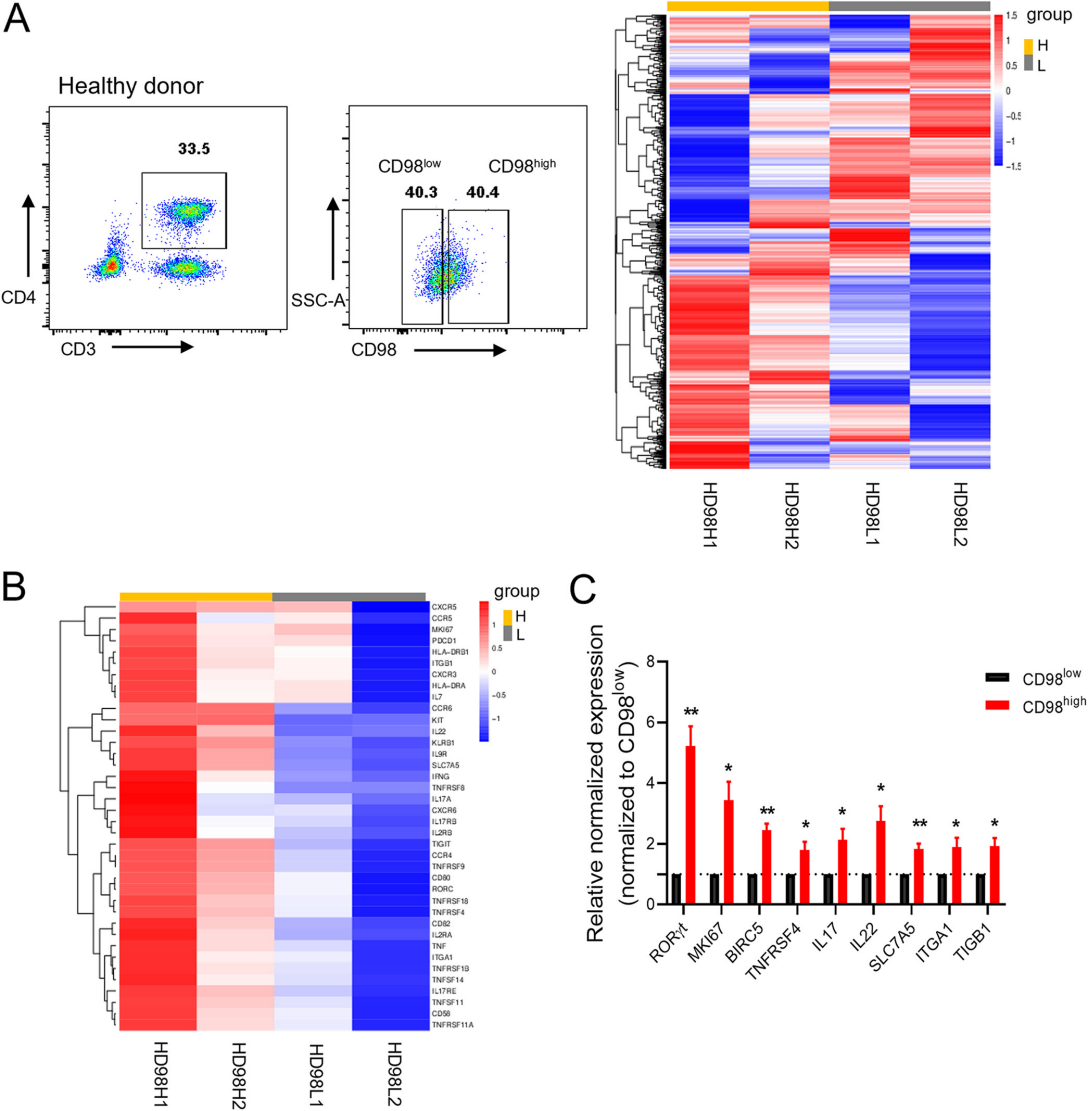
Distinct gene expression profile analyses of CD98^high^ CD4^+^ T cells. (A) Cell sorting strategies to gate CD98^low^ and CD98^high^ CD4^+^ T cells from PBMC of healthy donors. Representative flow cytometry plots are shown (left panel). The heat map demonstrates differentially expressed genes between CD98^high^ and CD98^low^ CD4^+^ T cells (adjusted *P* value, <0.05; fold change, ≥2) (right panel). (B) Genes associated with HIV infection among differentially expressed genes in the heat map are shown. CD98^high^ CD4^+^ T cells and CD98^low^ CD4^+^ T cells were purified by flow cytometry from PBMCs of two healthy donors. H, CD98^high^ CD4^+^ T cells; L, CD98^low^ CD4^+^ T cells; HD, healthy donor. (C) Verification of differential genes expression in CD98^high^ CD4^+^ T cells compared to that of CD98^low^ CD4^+^ T cells. CD98^high^ CD4^+^ T cells and CD98^low^ CD4^+^ T cells were purified by flow cytometry from HIV-1-infected individuals receiving cART (*n* = 5). The levels of mRNA expression were measured by RT-qPCR and normalized to that for *β-actin*. The dashed line indicates 1. The data represent the mean ± SD from triplicates. *P* values were calculated by the Mann-Whitney U test. *, *P < *0.05; **, *P < *0.01.

Next, we explored the characteristics of the CD98^high^ population. The CD98^high^ CD4^+^ T cells and CD98^low^ CD4^+^ T cells, which were defined by the 40th highest and lowest percentiles of CD98 fluorescence, respectively, were isolated from the PBMCs of two healthy donors by flow cytometry and subsequently analyzed by RNA sequencing (RNA-seq) (Fig. 3A). A total of 2,555 differentially expressed genes were identified in CD98^high^ CD4^+^ T cells and CD98^low^ CD4^+^ T cells. In CD98^high^ CD4^+^ T cells, 379 and 27 genes were upregulated and downregulated, respectively (adjusted *P* value, <0.05; fold change, ≥2) (Fig. S3A and B). Gene ontology analyses revealed that the upregulated genes in CD98^high^ CD4^+^ T cells are involved in various biological processes, including cytokine-cytokine receptor interactions, the NF-κB signaling pathway, and inflammatory response (Fig. S3C to F). Notably, an abnormal inflammatory response and immune activation can contribute to HIV-1 reservoir persistence by facilitating the proliferation and activation of HIV-1 infected cells (17).

10.1128/mbio.02496-22.3 FIG S3 Different cellular transcriptomic profile analysis in CD98^high^ CD4^+^ T cells compared to their CD98^low^ counterparts. (A and B) Venn diagrams of upregulated expressed genes (A) and downregulated genes (B) in CD98^high^ CD4^+^ T cells compared to their CD98^low^ counterparts. (C to F) Gene ontology analyses including KEGG pathway analysis (C) and the terms “biology process” (D), “cellular component” (E), and “molecular function” (F) enriched in upregulated genes among differentially expressed genes in CD98^high^ CD4^+^ T cells compared to their CD98^low^ counterparts. CD98^high^ CD4^+^ T cells and CD98^low^ CD4^+^ T cells were isolated from PBMCs of two healthy donors. Download FIG S3, TIF file, 1.0 MB. Copyright © 2022 Zhang et al. 2022 Zhang et al. https://creativecommons.org/licenses/by/4.0/ This content is distributed under the terms of the Creative Commons Attribution 4.0 International license.

In accordance with previous reports, these CD98 partner genes, such as *SLC7A5*, *ITGA1*, and *ITGB1*, were more highly expressed in CD98^high^ CD4^+^ T cells than in CD98^low^ CD4^+^ T cells. In addition, the upregulated genes in CD98^high^ CD4^+^ T cells included cytokine-cytokine receptor interaction-associated genes (*IL-17A*, *IL-22*, *TNFRSF4*, *CCR4*, *CCR5*, *CCR6*, and *CXCR3*), a transcription factor-associated gene (*RORγt*), and cellular proliferation-associated genes (*KIT* and *MKI67*) (Fig. 3B), especially, *KLRB1* (*CD161*), *TNFRSF8* (*CD30*), *TNFRSF4* (*OX40*), *HLA-DR*, *PDCD1* (*PD-1*), and *TIGIT*, which were previously reported to harbor more latent HIV-1 and were significantly upregulated in CD98^high^ CD4^+^ T cells (11, 18–21). Notably, TNFRSF4 and its downstream regulator, antiapoptotic protein BIRC5, can maintain the survival of the HIV-1 latent reservoir (22, 23). There was no significant difference between the two populations in terms of the expression of *CD32a*. According to a number of previous studies, whether CD32a acts as a bona fide biomarker of the HIV-1 latent reservoir is still controversial (9, 24, 25). The results of the RNA-seq analysis suggested that CD98^high^ CD4^+^ T cells might be important components of the HIV-1 reservoir.

To confirm the RNA-seq results, we performed an RT-qPCR assay to test the expression of the above-mentioned genes in CD98^high^ and CD98^low^ CD4^+^ T cells sorted by flow cytometry from HIV-1-infected individuals receiving cART, as described above. We found that the expression of CD98 partner genes (*SLC7A5*, *ITGA1*, and *ITGB1*), cytokine-associated genes (*IL-17A* and *IL-22*), a transcription factor-associated gene (*RORγt*), and cellular proliferation-associated genes (*KIT*, *MKI67*, *BIRC5*, and *TNFRSF4*) significantly increased in CD98^high^ CD4^+^ T cells, in contrast to those in CD98^low^ CD4^+^ T cells (Fig. 3C).

To investigate the variation of CD98 expression, we measured CD98 expression on CD4^+^ T cells from PBMCs of HIV-1-infected individuals receiving cART as well as healthy donors. The result showed that CD98 expression was significantly higher on CD4^+^ T cells from HIV-1-infected individuals receiving cART than those from healthy donors (Fig. S4A). To determine whether CD98 expression is due to gene expression random variation, we purified CD98^high^ or CD98^low^ CD4^+^ T cells from PBMCs of six healthy donors by flow cytometry. Subsequently, the two populations were cultured under IL-2, IL-7, or anti-CD3/CD28 for 14 days. Though all these culture conditions increased the CD98 expression on both CD98^high^ and CD98^low^ CD4^+^ T cells, we found that the expression levels of CD98 on previously sorted CD98^high^ cells remain persistently higher than those of their CD98^low^ counterparts under the same treatments (Fig. S4B). These results suggested that the discreet populations of CD98^high^ and CD98^low^ cells can be maintained after long-term culture and are not just random variation in gene expression.

10.1128/mbio.02496-22.4 FIG S4 Variation of CD98 expression on CD4 T cells. (A) The expression levels of CD98 on CD4^+^ T cells from PBMCs of HIV-1-infected individuals receiving cART (*n* = 7) (shown in orange) and healthy donors (*n* = 9) (shown in blue) were analyzed by flow cytometry. Representative histograms are shown. Fluorescence minus one (FMO) was used as a control. Student’s *t* test was used to calculate significance between the two groups. (B) CD98^low^ and CD98^high^ CD4^+^ T cells from PBMCs of healthy donors were sorted by flow cytometry and cultured with IL-2, IL-7, and anti-CD3/CD28 antibodies for 14 days. Then, the expression levels of CD98 were analyzed by flow cytometry (*n* = 6). MFI, mean fluorescence intensity. Data represent the mean ± SD. Bonferroni’s multiple-comparison test was used to determine the intergroup significance. *, *P < *0.05; **, *P < *0.01; ***, *P < *0.001; ****, *P < *0.0001. Download FIG S4, TIF file, 0.4 MB. Copyright © 2022 Zhang et al. 2022 Zhang et al. https://creativecommons.org/licenses/by/4.0/ This content is distributed under the terms of the Creative Commons Attribution 4.0 International license.

### The CD98^high^ CD4^+^ T cells are highly permissive for HIV-1 infection.

According to a previous study, the high expression of CD98 has been implicated in lymphocyte activation (26). Notably, CD98 was highly expressed in HCV-infected cells and responsible for promoting viral entry and propagation (27). Based on our RNA-seq analysis, we observed that T cell activation and exhaustion genes, such as *CCR5*, *HLA-DR*, *CD80*, *IL-2R*, and *PD-1*, were upregulated in CD98^high^ CD4^+^ T cells (Fig. 3B).

**FIG 4:**
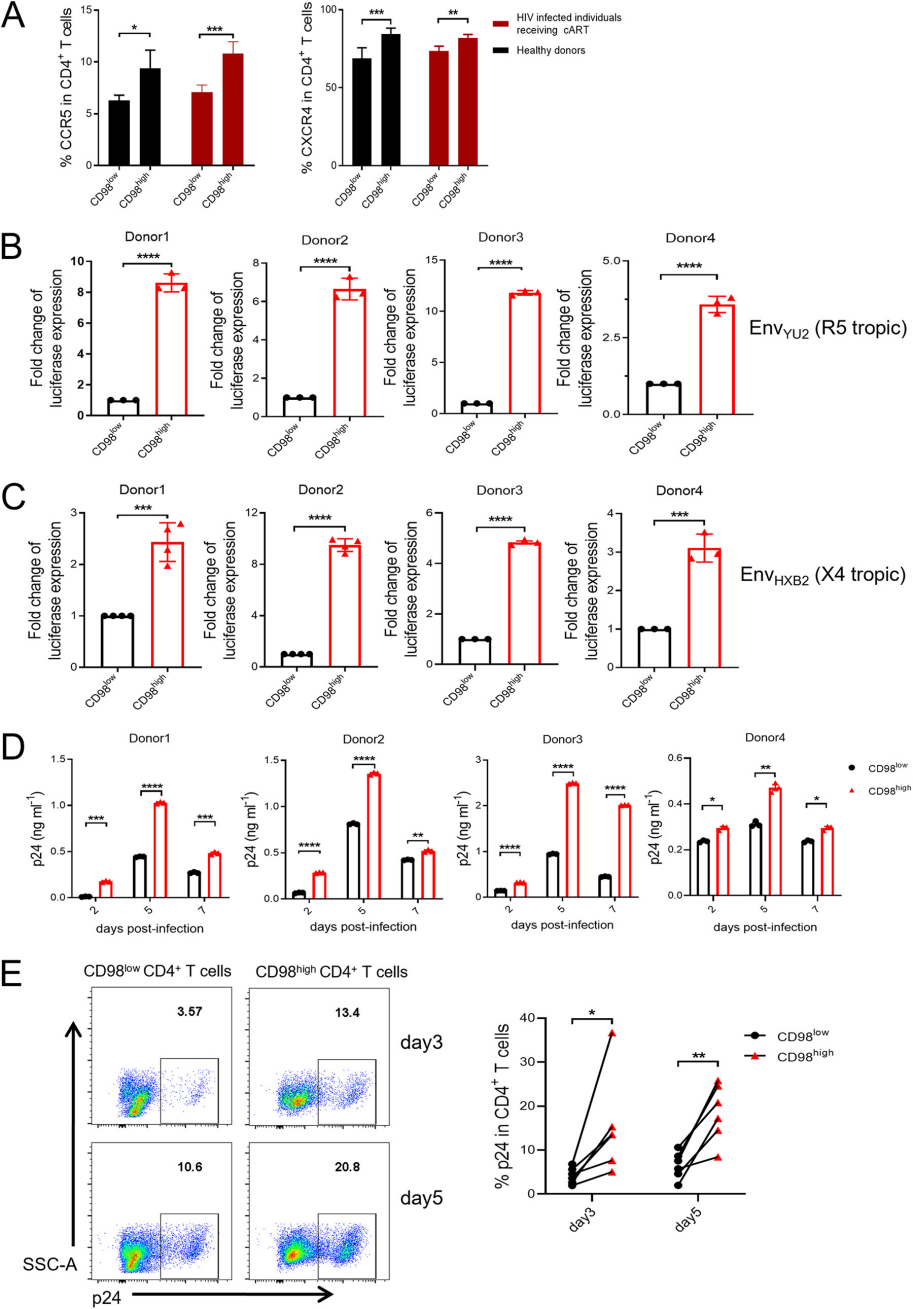
CD98^high^ CD4^+^ T cells are HIV-1 hyper-permissive cells. (A) The expression of CCR5 (left panel) and CXCR4 (right panel) on CD98^low^ and CD98^high^ CD4^+^ T cells from HIV-infected individuals receiving cART (shown in dark red) (*n* = 14) or healthy donors (shown in black) (*n* = 9). (B and C) CD98^low^ and CD98^high^ CD4^+^ cells were infected with Env_YU2_-luciferase (R5-tropic) (B) and Env_HXB2_-luciferase (X4-tropic) (C) pseudoviruses, respectively (pseudotyped with pHIV-luciferase with envelope from YU2 or HXB2, respectively). The luciferase activities were measured 72 h after infection (*n* = 4). (D and E) Infectivity of a wild-type HIV-1_NL4-3_ virus for CD98^low^ and CD98^high^ CD4^+^ T cells. The levels of HIV-1 p24 in supernatants were measured by enzyme-linked immunosorbent assay (ELISA) (*n* = 4) (D), and intracellular HIV-1 p24 levels were measured by flow cytometry (*n* = 6) (E) at the indicated time points postinfection. CD98^low^ and CD98^high^ CD4^+^ T cells were purified from PBMCs of healthy donors after TCR stimulation for 48 h. *P* values were calculated by Student’s *t* test or the Mann-Whitney U test. *, *P < *0.05; **, *P < *0.01; ***, *P < *0.001; ****, *P < *0.0001.

To further investigate the role of CD98 in HIV-1 permissiveness, we first determined the expression levels of HIV-1 coreceptors CCR5 and CXCR4 on CD98^low^ and CD98^high^ CD4^+^ T cells, respectively. The expression of CCR5 or CXCR4 was significantly higher in CD98^high^ CD4^+^ T cells than on their CD98^low^ counterparts from PBMCs of healthy donors as well as HIV-1-infected individuals receiving cART (Fig. 4A). These results suggest that CD98^high^ CD4^+^ T cells might be preferential targets for HIV-1 infection.

Next, we sorted CD98^high^ and CD98^low^ CD4^+^ T cells from the PBMCs of healthy donors using flow cytometry after stimulation with anti-CD3/CD28 antibodies for 48 h. Subsequently, a single-cycle infectivity assay was performed by infection with HIV-1 pseudoviruses, which were generated by cotransfecting HEK293T cells with an envelope-expressing plasmid, either pcDNA3.1-Env_Yu2_ (R5 tropic) or pcDNA3.1-Env_HXB2_ (X4 tropic), plus pHIV-1-luciferase and psPAX2. The infectivity of HIV-1 pseudoviruses was determined by measuring luciferase activity 72 h after infection. We observed that CD98^high^ CD4^+^ T cells had significantly higher luciferase activity than CD98^low^ CD4^+^ T cells, regardless of the presence of R5 tropic pseudoviruses or X4 tropic HIV-1 pseudoviruses (7.7-fold or 5.0-fold, respectively) (Fig. 4B and C). Moreover, these two populations were also infected with a wild-type HIV-1_NL4-3_ strain. HIV gag p24 was then measured postinfection in the supernatant or intracellularly at the indicated time points. We found that the expression levels of p24 in the supernatant or intracellularly were significantly higher in CD98^high^ CD4^+^ T cells than in their CD98^low^ counterparts (Fig. 4D and E). Overall, we confirmed that the susceptibility of CD98^high^ CD4^+^ T cells for HIV-1 infection increased significantly whether single-cycle or wild-type viral infection was implemented. Our results indicate that CD98^high^ CD4^+^ T cells are hyper-permissive for HIV-1 infection.

### CD98^high^ CD4^+^ T cells exhibit distinct immune phenotypes.

**FIG 5:**
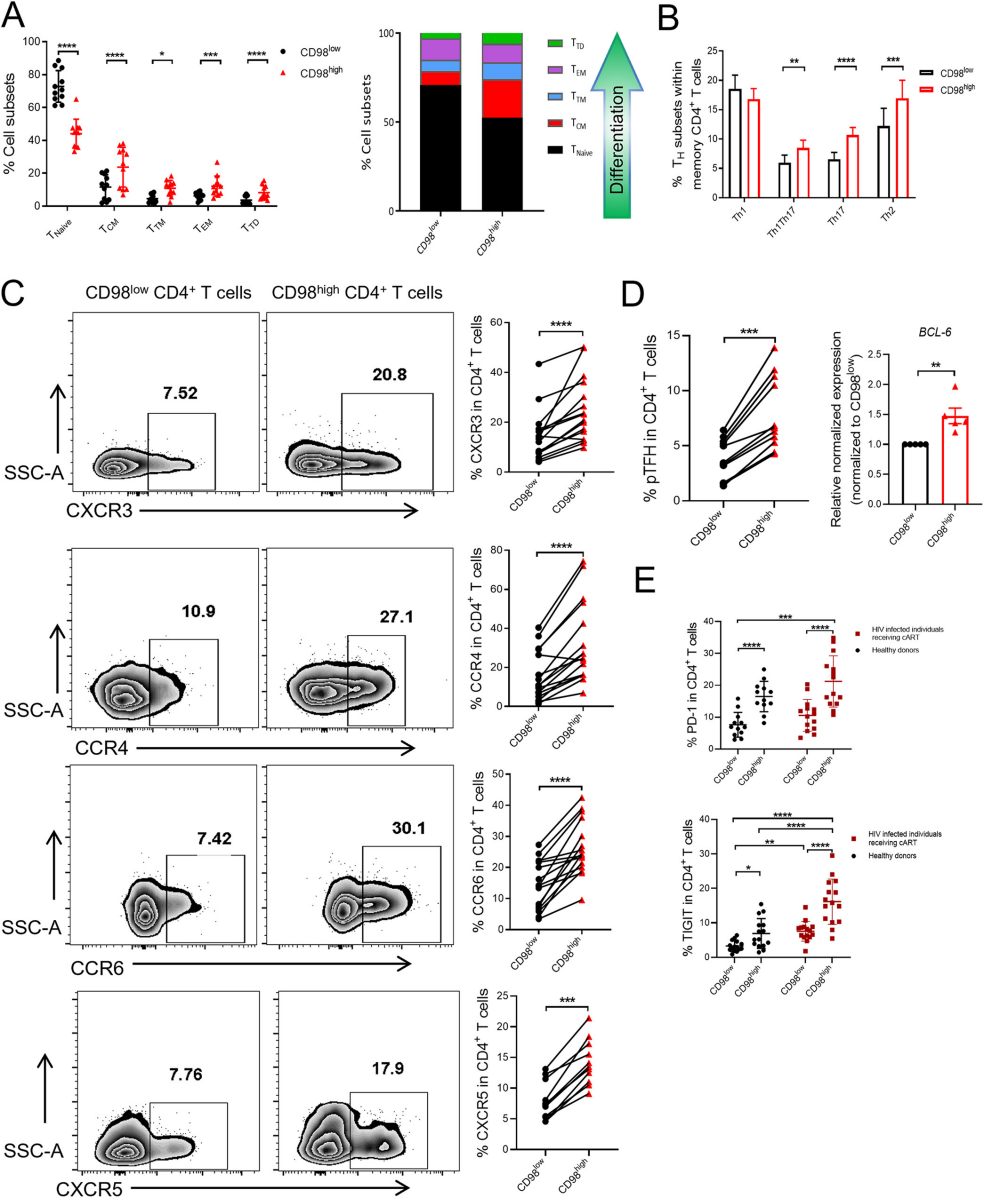
CD98^high^ CD4^+^ T cells possess distinct immune phenotypes. (A) Memory subset distribution in CD98^low^ and CD98^high^ CD4^+^ T cells from PBMCs of healthy donors (*n* = 11). T_Naive_, Naive T cell (CD45RO-); T_CM_, central memory T cell (CD45RO^+^ CD27^+^ CCR7^+^); T_TM_, transitional memory T cell (CD45RO^+^ CD27^+^ CCR7-); T_EM_, effector memory T cell (CD45RO^+^ CD27^–^ CCR7^–^); T_TD_, terminally differentiated memory cells (CD45RO^+^ CD27^–^ CCR7^+^). (B) The distribution of T helper subsets in CD98^low^ and CD98^high^ memory CD4^+^ T cells from PBMCs of healthy donors (*n* = 16). (C) The expression of chemokine receptors, including CXCR3, CCR4, CCR6, and CXCR5 on CD98^low^ and CD98^high^ CD4^+^ T cells from PBMCs of healthy donors (*n* = 16). Representative flow cytometry plots are shown. SSC-A, side scatter area. (D) The proportions of pTFH (PD-1^+^ CXCR5^+^) (*n* = 11) (left panel) and the expression level of *BCL-6* mRNA (*n* = 5) (right panel) in CD98^low^ and CD98^high^ CD4^+^ T cells from PBMCs of HIV-1 infected individuals receiving cART. (E) The expression of PD-1 (top panel) and TIGIT (bottom panel) on CD98^low^ and CD98^high^ CD4^+^ T cells from PBMCs of individuals receiving cART (shown in dark red) (*n* = 15) or healthy donors (shown in black) (*n* = 15). The data represent the mean ± SD. Student’s *t* test was used to calculate the significance between the two groups, and Bonferroni’s multiple-comparison test was used to determine the intergroup difference. *, *P < *0.05; **, *P < *0.01; ***, *P < *0.001; ****, *P < *0.0001.

According to previous reports, central memory CD4^+^ T cells are the major cellular reservoir for HIV-1 infection (28, 29). Therefore, we analyzed the memory phenotypes of CD98^high^ CD4^+^ T cells and CD98^low^ CD4^+^ T cells from PBMCs of healthy donors. The results showed that the proportions of memory CD4^+^ T cells, especially central memory CD4^+^ T cells (CD45RO^+^ CCR7^+^ CD27^+^), in CD98^high^ populations were significantly higher than those in CD98^low^ populations. Accordingly, the proportion of naive T cells decreased significantly in the CD98^high^ population compared to that in the CD98^low^ population (Fig. 5A). These results suggest that CD98^high^ CD4^+^ T cells might act as a major component of the HIV-1 latent reservoir.

Previous studies indicated that Th1Th17 or Th17 cells act as preferential target cells for HIV-1 persistence in individuals receiving cART (30, 31). CCR6 is a main cellular marker of Th17 and Th1Th17 cells, and CCR6^+^ CD4^+^ T cells had more integrated HIV-1 DNA than CCR6^–^ CD4^+^ T cells (32). Moreover, as reported recently, RORγt is a master transcription factor of Th17 cells that promotes HIV-1 gene expression and viral outgrowth (33). Both the RNA-seq and RT-qPCR results indicated that CD98^high^ CD4^+^ T cells expressed significantly higher levels of *CCR6*, *CCR4*, *RORγt*, *IL-22*, and *IL-17A* than those in CD98^low^ CD4^+^ T cells (Fig. 3B and C). Therefore, we further analyzed the distribution of T helper cell subsets, including Th1 (CXCR3^+^ CCR4^−^ CCR6^−^), Th2 (CXCR3^−^ CCR4^+^ CCR6^−^), Th1Th17 (CXCR3^+^ CCR4^−^ CCR6^+^), and Th17 (CXCR3^−^ CCR4^+^ CCR6^+^) cells in CD98^high^ memory CD4^+^ T cells and CD98^low^ memory CD4^+^ T cells. Our results showed that the proportions of Th17 and Th1Th17 cells were significantly higher in CD98^high^ memory CD4^+^ T cells than in CD98^low^ CD4^+^ T cells (Fig. 5B). Meanwhile, the expressions of CXCR3, CCR4, and CCR6 increased significantly in CD98^high^ CD4^+^ T cells compared to those in CD98^low^ CD4^+^ T cells (Fig. 5C). The results of flow cytometry analyses were consistent with the RNA-seq results and suggested that CD98^high^ CD4^+^ T cells exhibited characteristics of Th17 and Th1Th17 cells.

Previous studies revealed that peripheral follicular T helper cells are more susceptible to HIV-1 infection and are responsible for the persistence of the HIV-1 reservoir in HIV-1-infected individuals receiving cART (34–37). Therefore, using PD-1 and CXCR5 to characterize the pTFH cells in CD98^high^ CD4^+^ T cells or CD98^low^ CD4^+^ T cells, we found that the expression of CXCR5 and the proportion of pTFH cells were significantly higher in CD98^high^ CD4^+^ T cells than those in CD98^low^ CD4^+^ T cells from PBMCs of HIV-1-infected individuals receiving cART (Fig. 5C and D). Moreover, *BCL-6* is the primary transcription factor in TFH cells (38). The expression of *BCL-6* was also significantly higher in CD98^high^ CD4^+^ T cells than in CD98^low^ CD4^+^ T cells according to RT-qPCR data (Fig. 5D), which further verified that CD98^high^ CD4^+^ T cells were skewed to the pTFH cell phenotype.

Previous reports have shown that latent HIV-1 proviruses persist in T cells expressing exhausted markers, such as PD-1 (programmed cell death 1) and TIGIT (T cell immunoreceptor with Ig and ITIM domains), and the expression levels of PD-1 and TIGIT on CD4^+^ T cells are positively correlated with the size of the HIV-1 latent reservoir (39, 40). Therefore, we further verified that the expression of PD-1 or TIGIT was significantly higher on CD98^high^ CD4^+^ T cells than that on CD98^low^ CD4^+^ T cells from the PBMCs of HIV-1-infected individuals receiving cART as well as healthy donors using flow cytometry analyses (Fig. 5E).

Collectively, our results demonstrated that CD98^high^ CD4^+^ T cells primarily displayed a central memory CD4^+^ T cell phenotype with typical characteristics of Th17, Th1Th17, and pTFH cells. In addition, the expression of PD-1 or TIGIT was significantly higher in CD98^high^ CD4^+^ T cells than that in CD98^low^ CD4^+^ T cells. These results further suggest that the HIV-1 reservoir might preferentially enrich CD98^high^ CD4^+^ T cells.

### The CD4^+^ T cells with high levels of CD98 expression harbor more latent HIV-1.

**FIG 6:**
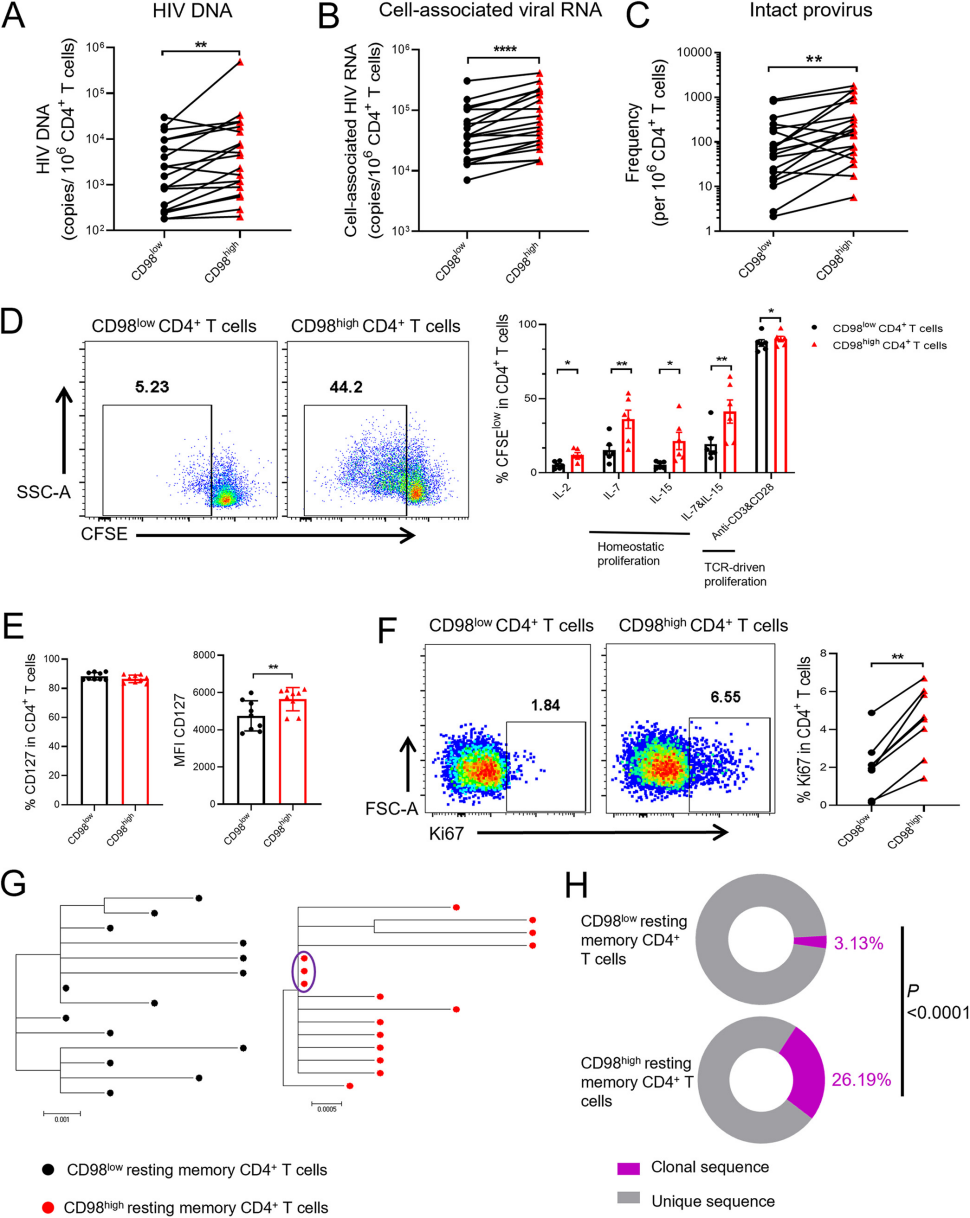
The CD4^+^ T cells with high levels of CD98 expression harbor more clonally expanded latent HIV-1. (A to C) HIV-1 DNA (*n* = 20) (A), cell-associated viral RNA (*n* = 20) (B), and intact HIV-1 provirus (*n* = 19) (C) were quantitated in CD98^low^ resting memory CD4^+^ T cells and CD98^high^ resting memory CD4^+^ T cells from PBMCs of HIV-1-infected individuals receiving cART. (D) CD98^low^ and CD98^high^ CD4^+^ T cells from PBMCs of HIV-1-infected individuals receiving cART (*n* = 6) were purified by flow cytometry and cultured with the indicated stimulus after being stained with CFSE, and CFSE dilution was measured after 7 days for stimulation. Representative flow cytometer plots showing the CFSE^low^ population within CD98^low^ or CD98^high^ CD4^+^ T cells with IL-7 stimulated at 7 days (left panel). SSC-A, side scatter area. (E) The expression of CD127 on CD98^low^ and CD98^high^ CD4^+^ T cells from PBMCs of HIV-1-infected individuals receiving cART (*n* = 9). MFI, mean fluorescence intensity. (F) The proportions of Ki67 in CD98^low^ or CD98^high^ CD4^+^ T cells from PBMCs of HIV-1 infected individuals receiving cART (*n* = 8). FSC-A, forward scatter area. Representative flow cytometer plots are shown. *P* values were calculated by the Mann-Whitney U test or Student’s *t* test. *, *P < *0.05; **, *P < *0.01; ***, *P < *0.001; ****, *P < *0.0001. (G) Representative phylogenetic trees of CD98^low^ resting memory CD4^+^ T cells and CD98^high^ resting memory CD4^+^ T cells from HIV-1-infected individuals receiving cART. Black dots, proviral sequences from CD98^low^ resting memory CD4^+^ T cells; red dots, proviral sequences from CD98^high^ resting memory CD4^+^ T cells; purple circle, identical clonal expansion sequences. (H) The percentages of clonal expansion proviral sequence within CD98^low^ resting memory CD4^+^ T cells and CD98^high^ resting memory CD4^+^ T cells from PBMCs of HIV-1-infected individuals receiving cART (*n* = 4). Purple symbols, percentage of clonal sequences; gray symbols, percentage of unique sequences. The *P* value was calculated by the chi-square test.

We found that CD98^high^ CD4^+^ T cells are hyper-permissive for HIV-1 infection and display distinct immune phenotypes. These characteristics implied that CD98^high^ CD4^+^ T cells might serve as an important component of the HIV-1 latent reservoir. Therefore, we next investigated whether CD98^high^ CD4^+^ T cells harbored more HIV-1 proviruses. We sorted CD98^high^ resting memory CD4^+^ T cells (CD3^+^ CD4^+^ CD45RO^+^ HLA-DR- CD98^high^) and their CD98^low^ counterparts from the PBMCs of HIV-1-infected individuals receiving cART (Fig. S5A) and compared the size of the viral reservoir by quantifying the total HIV-1 DNA, cell-associated viral RNA, and intact proviral DNA between the two populations. The results suggested that the expression levels of HIV-1 total DNA, cell-associated viral RNA, and intact proviral DNA were significantly higher in CD98^high^ resting memory CD4^+^ T cells than those in their CD98^low^ counterparts (Fig. 6A to C). Our results revealed that CD98^high^ resting memory CD4^+^ T cells harbor more latent HIV-1 than their CD98^low^ counterparts.

10.1128/mbio.02496-22.5 FIG S5 Phylogenetic analysis of proviral sequences from CD98^high^ CD4^+^ T cells and CD98^low^ CD4^+^ T cells. (A) Cell sorting strategy to gate CD98^high^ resting memory CD4^+^ T cells and CD98^low^ resting memory CD4^+^ T cells from PBMCs of HIV-1-infected individuals receiving cART. Representative flow cytometry plots were shown. (B) Phylogenetic trees of CD98^low^ resting memory CD4^+^ T cells and CD98^high^ resting memory CD4^+^ T cells from the other three HIV-1 infected individuals are shown. Black dots, proviral sequences from CD98^low^ resting memory CD4^+^ T cells; red dots, proviral sequences from CD98^high^ resting memory CD4^+^ T cells; purple circles, identical clonal expansion sequences. Download FIG S5, TIF file, 1.3 MB. Copyright © 2022 Zhang et al. 2022 Zhang et al. https://creativecommons.org/licenses/by/4.0/ This content is distributed under the terms of the Creative Commons Attribution 4.0 International license.

### CD98^high^ CD4^+^ T cells have higher proliferation potential than CD98^low^ CD4^+^ T cells in HIV-1-infected individuals receiving cART.

It has been reported that clonal expansion of HIV-1 latently infected cells plays an important role in the persistence of the viral reservoir (41, 42). Three principal factors were reported to be responsible for the expansion of the HIV-1 reservoir, including homeostatic proliferation, antigen-driven T cell proliferation, and integration site-driven proliferation (8). IL-7 and IL-15 are critical cytokines that maintain the homeostatic proliferation of memory CD4^+^ T cells (28, 43). Additionally, previous studies reported that CD98 is critical for the facilitation of lymphocytic proliferation depending on its integrin-binding domain, whereas the downregulation of CD98 impairs T cell clonal expansion (46, 47). To determine whether CD98 is involved in the expansion of the HIV-1 reservoir, we sorted CD98^high^ and CD98^low^ CD4^+^ T cells from the PBMCs of HIV-1-infected individuals receiving cART and stained them with carboxyfluorescein succinimidyl ester (CFSE, a dye indicating the cell division cycle). The two populations were stimulated with IL-7, IL-15, IL-7/IL-15, or anti-CD3/CD28 antibodies, respectively. The CFSE dilution was detected after 7 days of stimulation by flow cytometry. The results revealed that CD98^high^ CD4^+^ T cells had significantly higher proliferation than CD98^low^ CD4^+^ T cells in response to homeostatic cytokine signals or TCR stimulation (Fig. 6D). Although the proportions of CD127 (IL-7 receptor) were comparable between the two populations, the mean fluorescence intensity (MFI) of CD127 was significantly higher in CD98^high^ CD4^+^ T cells than in their CD98^low^ counterparts (Fig. 6E). In addition, the expression of the proliferation marker Ki67 has been reported to be associated with a larger size of the viral reservoir (28, 35). Accordingly, the proportion of Ki67 increased significantly in CD98^high^ CD4^+^ T cells compared to that in their CD98^low^ counterparts from PBMCs of HIV-1-infected individuals receiving cART (Fig. 6F). To further confirm whether CD98^high^ CD4^+^ T cells contribute to the HIV-1 reservoir clonal expansion, we isolated CD98^high^ resting memory CD4^+^ T cells and CD98^low^ resting memory CD4^+^ T cells from PBMCs of four HIV-1-infected individuals receiving cART as previously described (Fig. S5A). The single-genome proviral sequencing was then performed, and the HIV-1 *env* diversity lineages were analyzed. We found that the CD98^high^ resting memory CD4^+^ T cells from all four donors harbored more identical proviral *env* sequences, while CD98^low^ resting memory CD4^+^ T cells from only one donor harbored clonal proviral sequences. Representative phylogenetic trees of CD98^high^ resting memory CD4^+^ T cells and CD98^low^ resting memory CD4^+^ T cells are shown in Fig. 6G, and the results of other donors are shown in Fig. S5B. We found that 26.19% of the proviral sequence was clonal from CD98^high^ resting memory CD4^+^ T cells, while 3.13% of it was from the CD98^low^ counterparts (Fig. 6H). The summary of phylogenetic analyses of proviral HIV-1 *env* sequences is shown in Table 2. Overall, these results suggested that CD98^high^ resting memory CD4^+^ T cells harbored more clonal expansion proviral sequences and made a larger contribution to HIV-1 reservoir constitution than their CD98^low^ counterparts.

**TABLE 2:** Number of identical sequences obtained from CD98^low^ resting memory CD4^+^ T cells and CD98^high^ resting memory CD4^+^ T cells

Participant identifier	CD98^low^ resting memory CD4^+^ T cells			CD98^high^ resting memory CD4^+^ T cells		
	No. of sequences analyzed	No. of identical sequences	Clone rate (%)	No. of sequences analyzed	No. of identical sequences	Clone rate (%)
1	14	0	0	15	3	20
2	9	0	0	10	3	30
3	16	0	0	9	3	33.33
4	25	2	8	8	2	25
Total	64	2	3.13	42	11	26.19

Given that HIV-1 integration sites can drive latent reservoir proliferation as well (24, 46), we measured the HIV-1 integration sites of the CD98^high^ population and CD98^low^ population from J-mC cell lines. A total of 9,951 and 11,136 integration sites were obtained from the CD98^high^ population and CD98^low^ population, respectively. We found that chromosomes 19, 17, and 16 harbored more abundant integration sites than any other chromosomes, whether in the CD98^high^ population or CD98^low^ population (Fig. S6A). In addition, 58.33% and 59.36% of the integration sites were distributed on intron regions in the CD98^high^ population and CD98^low^ population, respectively (Fig. S6B and C). The distribution of integration sites was consistent with previous reports, and there was no significant difference between the two populations (16). Interestingly, the percentage of more than 16, especially 64 identical, integration sites in the CD98^high^ population was significantly higher than that of the CD98^low^ population (*P < *0.0001, chi-square test was used). Thus, clonally expanded viral integration sites in the CD98^high^ population were significantly higher than those in the CD98^low^ population (Fig. S6D). This further suggested that the CD98^high^ population in HIV-1 latently infected cells harbors more clonal expansion proviruses than the CD98^low^ population.

10.1128/mbio.02496-22.6 FIG S6 HIV-1 integration site distribution in CD98^high^ and CD98^low^ latently infected cells. (A) Comparison of the chromosome distribution of all integration sites between the CD98^high^ population (light green) and the CD98^low^ population (light gray). (B and C) The proportions of the integration site from the CD98^high^ population (B) and the CD98^low^ population (C) in the indicated genomic regions. (D) The proportion of identical HIV-1 integration sites from the CD98^high^ population and the CD98^low^ population. Purple, no fewer than 64 identical integration sites; orange, no fewer than 16 and fewer than 64 identical integration sites; blue, no fewer than 4 and fewer than 16 identical integration sites; black, fewer than 4 identical integration sites. The *P* value was calculated from the proportions of no fewer than 64 identical integration sites in the CD98^high^ population and the CD98^low^ population by the chi-square test. Download FIG S6, TIF file, 1.4 MB. Copyright © 2022 Zhang et al. 2022 Zhang et al. https://creativecommons.org/licenses/by/4.0/ This content is distributed under the terms of the Creative Commons Attribution 4.0 International license.

Taken together, our results demonstrate that CD98^high^ CD4^+^ T cells constitute a major component of the HIV-1 latent reservoir and possess a significantly higher clonal expansion capability that might contribute to the persistence of the latent HIV-1 reservoir.

## DISCUSSION

The identification of specific biomarkers of the HIV-1 reservoir is crucial for the immune surveillance system to recognize and eradicate HIV-1-expressing cells (6, 25). In this study, we showed that the expression of CD98 was higher in HIV-1 latently infected cells than in uninfected cells, and CD98^high^ CD4^+^ T cells are highly susceptible to HIV-1 infection. Further analysis suggests that memory CD4^+^ T cells, especially central memory CD4^+^ T cells, preferentially belong to CD98^high^ CD4^+^ T cells. In addition, CD98^high^ CD4^+^ T cells exhibited typical Th17 and Th1Th17 cell characteristics with increased *RORγt* and CCR6 expressions. Furthermore, there were higher proportions of pTFH cells within CD98^high^ CD4^+^ T cells than their CD98^low^ counterparts. Moreover, CD98^high^ resting memory CD4^+^ T cells harbored more intact proviruses than their CD98^low^ counterparts from HIV-1-infected individuals receiving cART. Most importantly, the CD98^high^ CD4^+^ T cells could significantly contribute to the expansion of the HIV-1 reservoir, as the evidence for robust proliferation potential and clonal expansion is quite solid. Therefore, CD98 may be a potential target for manipulation to reduce HIV-1 permissiveness, restrict the clonal proliferation of latently infected cells, and improve the treatment outcomes in HIV-1-infected individuals. Nevertheless, it has been well reported that the lymphoid tissue structures, including lymph nodes, gut mucosa, and even reproductive tracts, are the major HIV-1 reservoirs as well (11, 47, 48). Our results are limited to circulating CD4^+^ T cells because of the limitations of the current specimen-collection approaches. The relevance of CD98 and HIV-1 latency still needs to be confirmed in tissues, especially in secondary lymphoid tissues.

PD-1, TIGIT, and LAG3 are immune checkpoint receptors associated with T-cell activation/proliferation and exhaustion, and their expression has been linked to the enrichment of integrated HIV-1 proviral DNA in infected individuals during cART (39). We observed that the expression of PD1 or TIGIT was significantly higher on CD98^high^ CD4^+^ T cells than on CD98^low^ CD4 T cells. The CD4^+^ T cells expressing CD30 were reported frequently to harbor HIV-1 RNA (11). Our RNA-seq analysis verified that CD30 was highly expressed in CD98^high^ CD4^+^ T cells. Additionally, CD161^+^ CD4^+^ T cells constitute an important component of the HIV-1 latent reservoir and can act as a critical driving force for the clonal expansion of the HIV-1 reservoir (17). Our results suggest that the expression of CD161 was also higher in CD98^high^ CD4^+^ T cells than in CD98^low^ CD4^+^ T cells. Taken together, our findings indicate that high-level expression of CD98 could serve as a representative cellular surface protein among a series of HIV-1 infection biomarkers and facilitate a comprehensive understanding of the viral latency mechanism. Moreover, the evidence of previously identified cellular proteins as HIV-1 latency biomarkers, including CD2, CD30, PD-1, LAG-3, and TIGIT, is mainly based on the increased expression levels of HIV-1 DNA and/or RNA through quantitative PCR (qPCR) or reverse transcription PCR (RT-PCR), owing to technical limitations in the past. As these measurements always overestimate the size of the HIV-1 latent reservoir, the detection of intact proviruses or full-length HIV-1 proviral sequences should be considered for further validation (10, 49, 50). Unfortunately, none of the aforementioned biomarkers, including PD-1, TIGIT, CD30, CD161, and CD32a is able to identify all HIV-1 latently infected cells. Nonetheless, these studies have revealed the highly heterogeneous and complicated nature of the HIV-1 latent reservoir (25, 51).

CD98 plays an essential role in many biological processes through its distinct domains. CD98 combined with light chains (LAT-1 or LAT-2) promotes the import of essential amino acids, which, in turn, can activate the mTOR pathway, leading to protein synthesis, promotion of cell growth, and proliferation (48–50). The amino acid metabolism as a critical part of cellular metabolism is mostly regulated by metabolic pathways that affect the function of immune cells. Previous studies showed that metabolic programming can affect the differentiation and function of CD4^+^ T cells, which consequently had an impact on their susceptibility to HIV-1 and HIV-1 latent reservoir establishment (52–54). Recently, a study suggested that amino acid metabolites can act as noninvasive plasma biomarkers that are associated with time to viral rebound after cART treatment interruption. For example, l-glutamic acid in plasma can predict a longer time to viral rebound in HIV-1-infected individuals (55). Moreover, the dysfunction of tryptophan metabolism promoted HIV-1 infection and reactivation and was associated with deteriorative HIV-1 pathogenesis (56). HIV-1 preferentially infected high metabolic CD4^+^ T cells with enhanced glycolysis and high oxidative phosphorylation (57). The complex interrelationship between immunometabolism, especially amino acid metabolism, and HIV-1 reservoir persistence and clonal expansion as an intriguing area of research remains to be revealed.

CD98 not only serves as a partner of amino acid transporters involved in amino acid metabolism, but also mediates integrin-dependent signals by interacting with integrin (ITGA1 or ITGB1), which contributes to cell proliferation, migration, and protection from cell apoptosis (51). In view of its distinct structure and function, CD98 plays a crucial role in various diseases. High levels of CD98 expression in cancer cells have been widely reported as a promising biomarker of various cancers and are correlated with a poor prognosis (58–60). In addition, CD98 participates in autoimmune diseases such as type I diabetes and encephalomyelitis by promoting the clonal expansion of lymphocytes (44, 45, 61). The blockade of CD98 in T cells can attenuate T cell migration, thereby contributing to the acceptance of cardiac allografts (62). Recently, Hasegawa identified R8H283 as a monoclonal antibody that specifically recognized the CD98 heavy chain protein as a prospective candidate in treating multiple myeloma (63). Hence, it is not unforeseeable that CD98 blockade may provide promising therapy approaches for various diseases in clinical trials. Additionally, CD98 is required for lymphocyte proliferation and activation, which is consistent with our RNA-seq analysis showing that a higher level of CD98 expression is strongly associated with T cell proliferation. Of note, CD98 plays an essential role in the clonal expansion of lymphocytes, as reported in several studies (44, 45, 64, 65).

The results of our study suggest that the CD98^high^ CD4^+^ T cells exhibit increasing proliferation abilities in response to homeostatic cytokine signals, including IL-7 and IL-15, which could potentially facilitate HIV-1 latent reservoir persistence. Notably, the CD98^high^ CD4^+^ T cells not only harbored more latent HIV-1 than their CD98^low^ counterparts, including HIV-1 DNA, cell-associated viral RNA, and intact provirus, but also possessed a high proportion of clonally expanded proviral sequences. Together, the CD98^high^ CD4^+^ T cells have a robust proliferative capability that significantly contributes to the persistence and the clonal expansion of the HIV-1 reservoir. It may be a novel approach that target CD98 by specific therapeutic antibodies, in combination with anti-gp120 antibody or chimeric antigen receptor T cell immunotherapy, inhibits the clonal expansion of latently infected CD4^+^ T cells and reduces the HIV-1 reservoir.

In line with other reports, HIV-1 latently infected cells exhibit a different expression profile compared to uninfected cells. Besides CD98, our proteomic analysis also found other interesting plasma membrane proteins (e.g., CD81, CD71, and BST2) that were highly expressed on HIV-1 latently infected cells. Based on their known function and signaling pathways, these proteins could be further explored in the HIV-1 latent reservoir. Despite the involvement of these proteins in HIV-1 infection, their role in HIV-1 latency infection remains unknown. Other plasma membrane proteins that have significantly higher expression in HIV-1 latently infected cells merit being further investigated, although these approaches could be limited, as no commercial antibodies are available for their detection by flow cytometry. In order to develop effective therapeutic strategies and achieve functional HIV-1 cures, it is worth investigating and analyzing comprehensively the differentially expressed proteins in HIV-1 latently infected cells and their potential to recognize viral the reservoir in different statuses.

## MATERIALS AND METHODS

### Participants.

This study was approved by the ethics review boards of Sun Yat-Sen University and the Eighth People’s Hospital of Guangzhou, Guangdong, China. Peripheral blood mononuclear cells from healthy blood donors were provided by the Shenzhen Blood Center. The HIV-1-infected participants were recruited on the basis of cART with plasma HIV-1 viremia below the limit of detection of standard clinical assays (less than 50 copies HIV-1 RNA mL ^−1^) and high CD4^+^ T cell count (at least 350 cells μL^−1^) for a minimum of 6 months. All human samples were anonymously coded in accordance with the local ethical guidelines (as stipulated by the Declaration of Helsinki). All participants were given written informed consent with approval of the institutional review board of Guangzhou Eighth People’s Hospital (Guangzhou, China).

### Cell lines.

HEK293T cells and Jurkat T cells were obtained from ATCC. HEK293T cells were cultured in conditioned Dulbecco’s modified Eagle’s medium (DMEM; Gibco, Invitrogen, Carlsbad, CA) containing 10% fetal bovine serum (FBS) (Gibco, Invitrogen) and 1% penicillin-streptomycin (Gibco, Invitrogen). Jurkat T, J-Lat 8.4, J-Lat 10.6, and J-mC cell lines were cultured in RPMI 1640 (Gibco, Invitrogen) containing 10% FBS and 1% penicillin-streptomycin. J-Lat 8.4 and J-Lat 10.6 cell lines were derived from the Jurkat T cells and were gifts from the Robert F. Siliciano (Department of Medicine, Johns Hopkins University School of Medicine, Baltimore, MD) laboratory and were originally generated by Eric Verdin (The Buck Institute for Research on Aging, Novato, CA); the J-mC cell line was generated by Kai Deng (Sun Yat-Sen University). All the cell lines were mycoplasma-free and cultured in an environment of 37°C and 5% CO_2_.

### Isolation and culture of primary CD4^+^ T cell populations.

The PBMCs derived from healthy donors or HIV-1 chronically infected individuals receiving cART were isolated by Ficoll-Hypaque gradient separation. Primary CD4^+^ T cells were obtained from PBMCs through negative magnetic selection with the human CD4 T lymphocyte enrichment set DM (BD IMag cell separation system) following the manufacturer’s protocol. The CD4^+^ T cells were cultured in RPMI 1640 supplemented with 10% FBS, 2 mM GlutaMAX (Gibco, Invitrogen, Carlsbad, CA), 1% penicillin-streptomycin, and 10 ng mL^−1^ recombinant human interleukin-2 (R&D Systems).

### Proteomic analysis of mass spectrometry.

About 10 million cells from J-Lat 8.4, J-Lat 10.6, and Jurkat T cells were lysed by sonication. Subsequently, protein quantitation was conducted by bicinchoninic acid (BCA) assay (Thermo Fisher Scientific). Then, 200 μg protein was extracted with 8 M urea in 0.1 M Tris base by 10 kDa ultra-filtration device (Merck Millipore) and reduced by 10 mM dithiothreitol for 60 min and then alkylated by 20 mM iodoacetamide (Thermo Fisher Scientific) for 30 min in the dark. Each sample was incubated with 2 μg trypsin at 37°C overnight for digestion. Next, digested peptides were extracted with 50 mM NH_4_HCO_3_ containing 0.4% TFA (trifluoroacetic acid) and subjected to vacuum for 3 h until the solvent was removed. Digested peptides were redissolved into 50% acetonitrile containing 0.1% TFA though C_18_ ZipTip (Millipore), followed by vacuum to remove the solvent again. Samples were stored at −80°C until the next step. Finally, proteolytic digests were dissolved into 0.01% formic acid and subjected for nanoscale LC-MS/MS with an EASY-nLC system connected to a Q-Exactive device with higher collisional dissociation fragmentation (Thermo Fisher Scientific) to characterize the differentially expressed proteins among the cell lines as previously described (66). The LC-MS/MS data were analyzed with PEAKS 6 Studio software (BSI, Canada).

### Flow cytometry and cell sorting.

The green fluorescent protein (GFP) fluorescence was measured in a BD LSR Fortessa cell analyzer and sorted with a FACSAria II instrument (BD Biosciences). The CD98^high^ and CD98^low^ CD4^+^ T cells were sorted by use of flow cytometry upon staining with CD3, CD4, and CD98 surface-staining antibodies. The purity of subsets was greater than 95%. Cells were stained with monoclonal antibodies of the surface markers for 30 min in the dark and then fixed by the use of fixation/permeabilization buffer (BD Biosciences), followed by intracellular cytokine or HIV-1 Gag p24 or Ki67 staining with antibodies directed against intracellular antigen. Details on the antibodies used for flow cytometry are provided in Table S2 in the supplemental material, and dead cells were excluded with fixable viability dye (eBioscience). Data were analyzed using FlowJo software (version 10.4.0).

10.1128/mbio.02496-22.8 TABLE S2 Antibodies for flow cytometry analysis. Download Table S2, DOCX file, 0.02 MB. Copyright © 2022 Zhang et al. 2022 Zhang et al. https://creativecommons.org/licenses/by/4.0/ This content is distributed under the terms of the Creative Commons Attribution 4.0 International license.

### Establishment of a cell line model of HIV-1 latency.

The cell line model of HIV-1 latency infection was established as previously described (12). Briefly, the pNL4-3-ΔEnv/ΔNef-d2EGFP pseudotyped HIV-1 construct (the *Env* gene is a frameshifted mutation, and the *GFP* gene encoding green fluorescent protein was inserted into the *Nef* gene as a fluorescent reporter gene; modifications were based on the HIV_NL4-3_ strain genome) was used to establish a Jurkat T cell latency infection model (the genomic structure is provided in Fig. S2A). HEK293T cells were cotransfected with 5 μg of vesicular stomatitis virus G (VSV-G) constructs and 15 μg of pseudovirus constructs. Then, 48 h posttransfection, pseudoviruses within supernatant were concentrated by polyethylene glycol (PEG) 6000. About one million Jurkat T cells were infected with pseudoviruses. On day 3, the GFP-positive population was sorted using a FACSAria II cell sorter (BD Biosciences) and cultured for another week to recover. After another 7 days, GFP-negative cells were sorted out. After cell sorting, these infected cells were >99% GFP negative (details of establishment are provided in Fig. S2B). Upon tumor necrosis alpha (TNF-α) or other HIV latency reversal agent stimulation, these GFP-negative cells were reactivated and became GFP positive (Fig. S2C). We named this Jurkat T latency cell line J-d2E-GFP- and it was used for evaluating the latency biomarker.

### Establishment of a primary CD4^+^ T cell model of HIV-1 latency *in vitro*.

The latently infected CD4^+^ T cell model was established as previously described with a few modifications (10). The isolated CD4^+^ T cells from healthy donors were stimulated with anti-CD3 antibody at 1 μg m1^−1^ (R&D Systems), anti-CD28 antibody at 1 μg m1^−1^ (R&D Systems), and IL-2 at 10 ng mL^−1^ (R&D Systems). Then, 48 h after stimulation, the cells were infected with HIV_NL4-3-GFP_ (a viral strain with GFP after Nef is separated by P2A). After infection (1 week), the cells were cultured in medium supplemented with IL-2 at 10 ng mL^−1^ (R&D Systems) and then maintained in a resting station with IL-7 at 1 ng mL^−1^ (R&D Systems) for another week. On days 0, 7, and 14, cells were harvested and analyzed by flow cytometry for the expression of GFP or p24 and CD98. Dead cells were excluded with fixable viability dye (eBioscience) following the manufacturer’s protocol. Data were analyzed using FlowJo software (version 10.4.0).

### Real-time RT-qPCR analysis.

Total RNA was isolated with TRIzol reagent (Life Technologies) and then subjected to cDNA synthesis using a HiScript II 1st-strand cDNA synthesis kit (Vazyme) following the manufacturer’s protocol. The gene-specific primer sequences used for real-time RT-qPCR analysis are listed in Table S3 in the supplemental material. Quantitative PCR was performed with SYBR qPCR master mix (Vazyme). The 2^–△△^*^CT^* method was adopted to analyze the relative expression of mRNA, using *β-actin* as the reference gene.

10.1128/mbio.02496-22.9 TABLE S3 Gene-specific primer sequences. Download Table S3, DOCX file, 0.02 MB. Copyright © 2022 Zhang et al. 2022 Zhang et al. https://creativecommons.org/licenses/by/4.0/ This content is distributed under the terms of the Creative Commons Attribution 4.0 International license.

### RNA-seq analysis.

A total of 1 to 2 million CD98^high^ and CD98^low^ CD4^+^ T cells from PBMC of two healthy donors were sorted by flow cytometry. Total RNA was extracted using TRIzol (Invitrogen) following the manufacturer’s protocol. The four libraries were sequenced on the Illumina NovaSeq 6000 platform based on sequencing by synthesis with 100-bp paired-end reads. The sequencing reads from all RNA-seq data were aligned to the human (hg18) reference genome. Differential gene expression analyses were performed using a fold change of ≥2 and a *P* of ≤0.05 as the cutoff. The sequencing was performed at the Annoroad Genomics Technology Institute (Beijing, China).

### Luciferase reporter assays.

HEK293T cells were cotransfected with 7.5 μg pcDNA3.1-Env_HXB2_ or pcDNA3.1-Env_YU2_ coding HIV-1X4 tropic or R5 tropic envelope, 10 μg psPAX2, which is a packaging plasmid, and 10 μg pHIV-luciferase using a polyethylenimine transfection system and following the manufacturer’s instructions to produce HIV-Env_HXB2_ (derived from pcDNA3.1-Env_HXB2_)-luciferase or HIV-Env_YU2_ (derived from pcDNA3.1-Env_YU2_)-luciferase virus, respectively. Supernatants were harvested after 48 h and filtered through 0.45-μm filters. The CD98^high^ and CD98^low^ CD4^+^ T cells from healthy donors were sorted and infected with HIV-luciferase virus poststimulated for 48 h with IL-2 at 10 ng mL^−1^, anti-CD3 antibody at 1 μg m1^−1^, and anti-CD28 antibody at 1 μg m1^−1^. Luciferase activities in the cell lysates were measured through a luciferase reporter assay system (Promega) after 48 to 72 h infection. Fold changes were normalized by comparison to levels for the CD98^low^ population according to light units.

### Quantification of HIV-1 proviral DNA and intracellular RNA.

CD98^high^ and CD98^low^ resting memory CD4^+^ T cells from PBMCs of HIV-infected individuals receiving cART were sorted by a FACSAria II cell sorter (BD Biosciences) upon staining with CD3, CD4, CD98, CD45RO, and HLA-DR surface-staining antibodies.

Sorted populations were subjected to DNA and RNA extraction using commercial kits purchased from Magen (catalog number R5111-02) following the manufacturer’s instructions. The expressions of HIV-1 proviral DNA were determined for qPCR using Gag primers and probe (18).

The quantitation of HIV-1 cell-associated RNA, a specific reverse primer, was used to reverse-transcribe HIV-1 RNA. The qPCR assay was performed for specific reverse-transcribed HIV-1 cDNA with HIVTot RNA primer pairs (67, 68). After quantitation, an *in vitro* transcribed HIV-1 RNA was used as the external control for measuring cell-associated viral RNAs. An HIV-1 RNA transcribed *in vitro* was used as the external control. The threshold cycle (*C_T_*) values of each sample were converted to mass and further converted to the number of HIV-1 RNA copies. The final expression of intracellular HIV-1 RNAs was represented as 10^3^ copies of viral RNA per million CD4^+^ T cells for each sample.

### Intact proviral DNA assay (IPDA).

CD98^high^ resting memory CD4^+^ T cells (CD3^+^ CD4^+^ CD45RO^+^ HLA-DR^–^ CD98^high^) and their CD98^low^ counterparts (CD3^+^ CD4^+^ CD45RO^+^ HLA-DR^–^ CD98^low^) from the PBMCs of HIV-1-infected individuals receiving cART were sorted using a FACSAria II cell sorter. We extracted genomic DNA from the two populations with precautions to avoid excess DNA fragmentation using the AllPure total DNA/RNA micro kit (Magen).

The HIV intact proviral DNA value was the total of intact and defective proviruses determined with minor modifications by droplet digital PCR (ddPCR) (Bio-Rad Laboratories). The experiment consists of two independent hydrolysis probe reactions targeting different conserved regions of the HIV genome (1, 69). Briefly, one reaction targets the viral packaging signal (ψ) region with the amplicon positioned at HXB2 coordinates 692 to 797. The reaction used a 5′-FAM-labeled hydrolysis probe. The amplification of the ψ region produces a FAM fluorescence signal. The other reaction targets the envelope region of the proviral genome, with the amplicon positioned at HXB2 coordinates 7736 to 7851. This reaction contains two hydrolysis probes: a 5′-VIC-labeled probe specific for wild-type proviral sequences and a 5′-unlabeled competitor probe specific for APOBEC-3G/H hypermutated sequences. The successful amplification of a wild-type envelope region produces a VIC fluorescence signal, while the amplification of a hypermutated form cannot produce a fluorescence signal. Droplets containing intact proviral DNA are positive for both FAM and VIC fluorescence signals. Droplets containing 3′ defective or hypermutated proviral DNA are positive for only an FMA fluorescence signal. Droplets containing 5′ defective proviral DNA are positive for only VIC fluorescence signal. Droplets containing few proviral DNAs are negative for both FMA and VIC fluorescence signals. Meanwhile, quantification of DNA shearing from the two cell populations was performed using two independent hydrolysis probe reactions that interrogate the human *RPP30* gene. Then, the DNA shearing index from the *RPP30* gene was calculated to correct the raw ddPCR output of intact proviruses. After correction, the results were shown as the frequency of intact proviruses per 10^6^ CD4^+^ T cells.

### Single-genome proviral sequencing and phylogenetic analysis.

Genomic DNA was extracted from the CD98^high^ resting memory CD4^+^ T cells and their CD98^low^ counterparts from four HIV-1-infected individuals receiving cART. Nested PCR was performed to amplify the HIV-1 *env* regions (including V1 to V3) using *Taq* high-fidelity polymerase (Vazyme) (1). Briefly, the first-round PCR program was 94°C for 2 min and then 35 cycles of 94°C for 15 s, 55°C for 30 s, and 72°C for 2 min, followed by 72°C for 10 min. The second-round PCR program was 94°C for 2 min and then 35 cycles of 94°C for 15 s, 55°C for 30 s, and 72°C for 1 min, followed by 72°C for 10 min. Then, 2 μL of the PCR product from the first round was used as the second round PCR template. The second-round PCR products were run on a precast 1% agarose gel (Bioweste). The PCR products were ligated into pMD-18 T vector (TaKaRa). Then, single clones were picked from each population and proceeded to Sanger sequencing. The sequences from each population were aligned using MUSCLE, and sequences with ambiguous positions were removed. The phylogenetic trees were generated using the maximum likelihood method with 1,000 bootstrap replications implemented with MEGA 7.

### High-throughput HIV-1 integration site analysis.

A total of 1.5 to 2 million CD98^high^ and CD98^low^ J-mC cells were sorted by flow cytometry. Genomic DNA was isolated according to the manufacturer’s protocol (blood and cell culture DNA minikit, Qiagen) and sheared by sonication (Covaris, Woburn, MA) to obtain 300- to 500-bp random fragments for processing.

The sheared DNA fragments were end-repaired using the End-It DNA end repair kit (Epicentre, Madison, WI), and a single deoxyribosyladenine (dA) was added to the 3′ ends using a dA-tailing kit (NEB, Ipswich, MA) following the manufacturer’s protocol. A partially double-stranded linker with the 3′ dA overhang on the genomic DNA fragments and the 3′ T overhang on the linker DNA prevented genomic-genomic DNA and linker-linker ligation, increased the efficiency of the desired ligation, and reduced the artifacts associated with cross-ligation commonly seen with restriction enzyme-based linker-mediated PCR (LM-PCR). Then, excess linkers and short DNA products were removed using the Agencourt AMPure XP system (Beckman Coulter).

In preparation for DNA library, the integration junctions were selectively amplified using one primer that matched the HIV long terminal repeat (LTR) sequence and the other primer that matched the single-stranded portion of the linker. Then, excess linkers and short DNA products were removed using the Agencourt AMPure XP system (Beckman Coulter) once again. Nested PCR was performed with an inner primer pair; one primer matched the sequence of the LTR and the other primer matched the linker. Then, the second PCR amplification increased the specificity of amplification and was used to introduce the Illumina sequencing adapters and barcodes (sequences of the primers and linkers for integration site analysis shown in Table S4).

10.1128/mbio.02496-22.10 TABLE S4 Sequences of the primers and linkers for integration site analysis. Download Table S4, DOCX file, 0.01 MB. Copyright © 2022 Zhang et al. 2022 Zhang et al. https://creativecommons.org/licenses/by/4.0/ This content is distributed under the terms of the Creative Commons Attribution 4.0 International license.

### Statistical analyses.

Data are presented as the mean ± standard deviations (SD) from at least triplicates. For data with a normal distribution, we used Student’s *t* test, and otherwise a nonparametric exact Wilcoxon signed-rank test was used. For multiple comparisons, a one-way or two-way analysis of variance (ANOVA; for parametric data) followed by Bonferroni’s correction (only two groups were compared) or Dunnett’s test (all groups were compared to one control group). The chi-square test was used to analyze the proportions of clonal expanded sequences and identical integration sites. A *P* value of <0.05 was considered statistically significant and denoted by *, while *P* values of <0.01 were denoted by **, *P *values of <0.001 were denoted by ***, and *P* values of <0.0001 were denoted by ****. Statistical analyses were performed using GraphPad Prism 8.0 (GraphPad).

### Data availability.

All data needed to evaluate the conclusions in the paper are described in the paper and/or the supplemental material.
