# Supplementary figures and images for: Identification of CD98 as a Novel Biomarker for HIV-1 Permissiveness and Latent Infection
Source: mBio. 2022 Oct 10;13(6):e02496-22. doi: 10.1128/mbio.02496-22 (PMC9765422; doi:10.1128/mbio.02496-22)

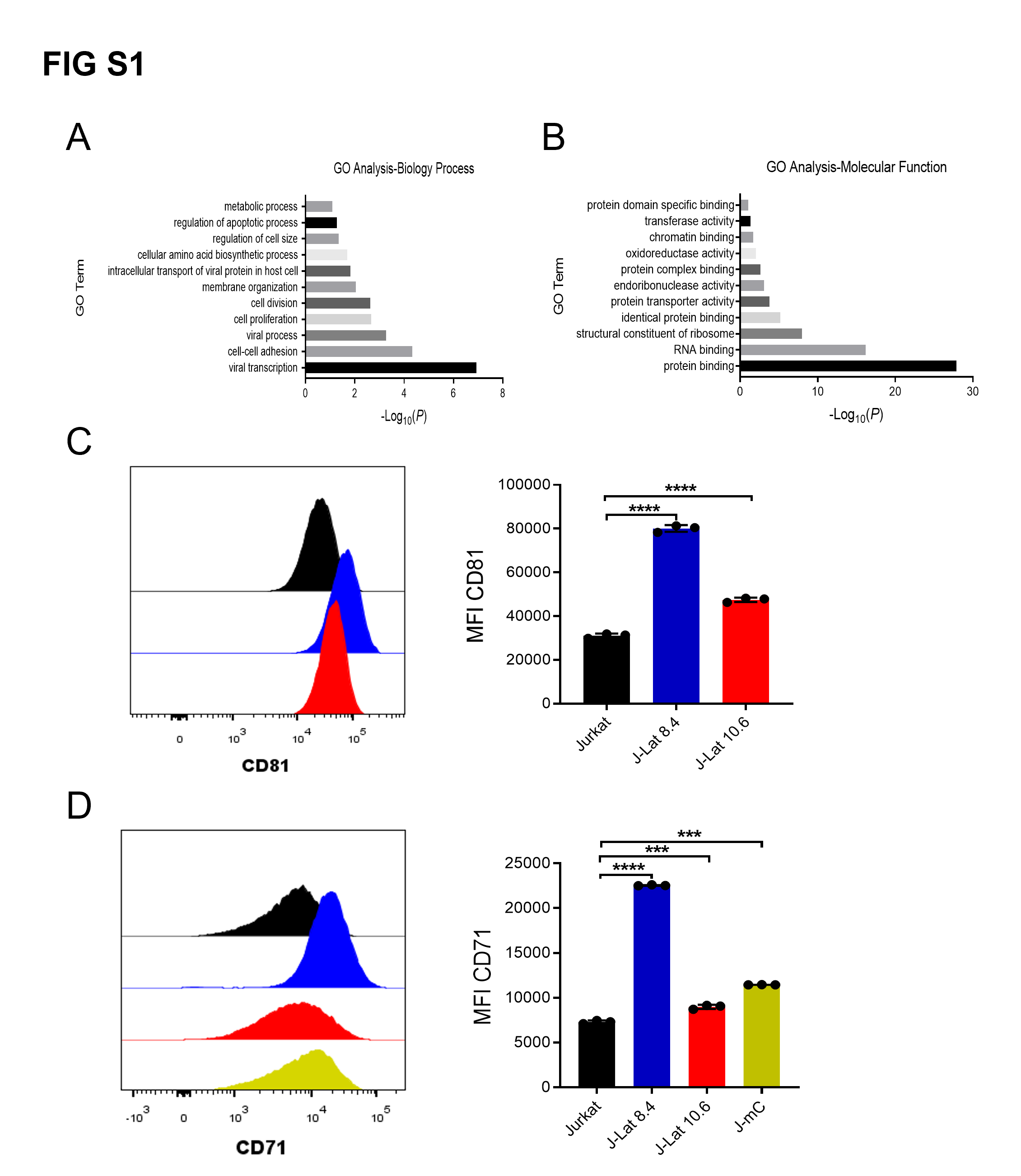

Supplement: FIG S1 [file mbio.02496-22-s0001.tif]

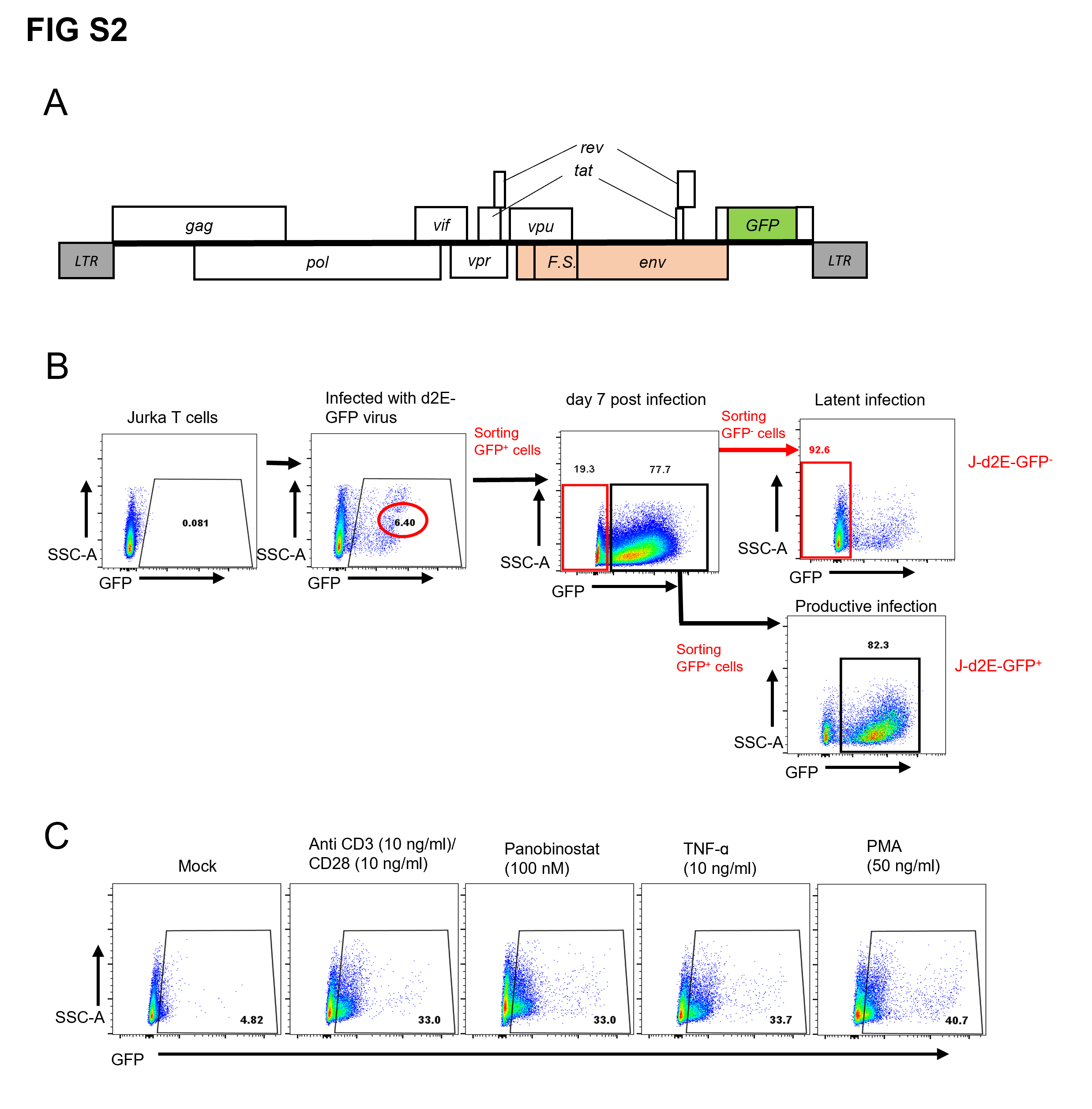

Supplement: FIG S2 [file mbio.02496-22-s0002.tif]

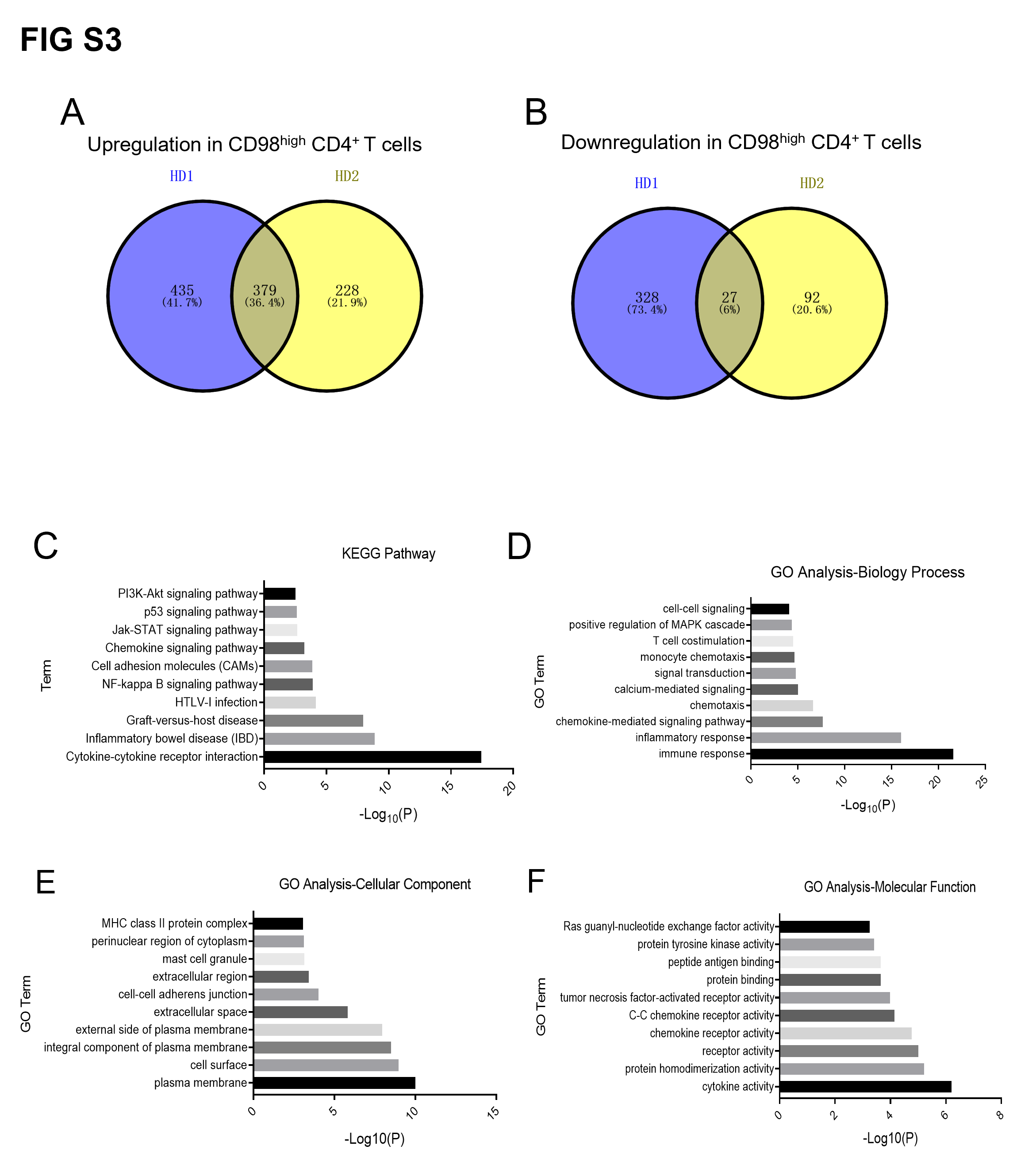

Supplement: FIG S3 [file mbio.02496-22-s0003.tif]

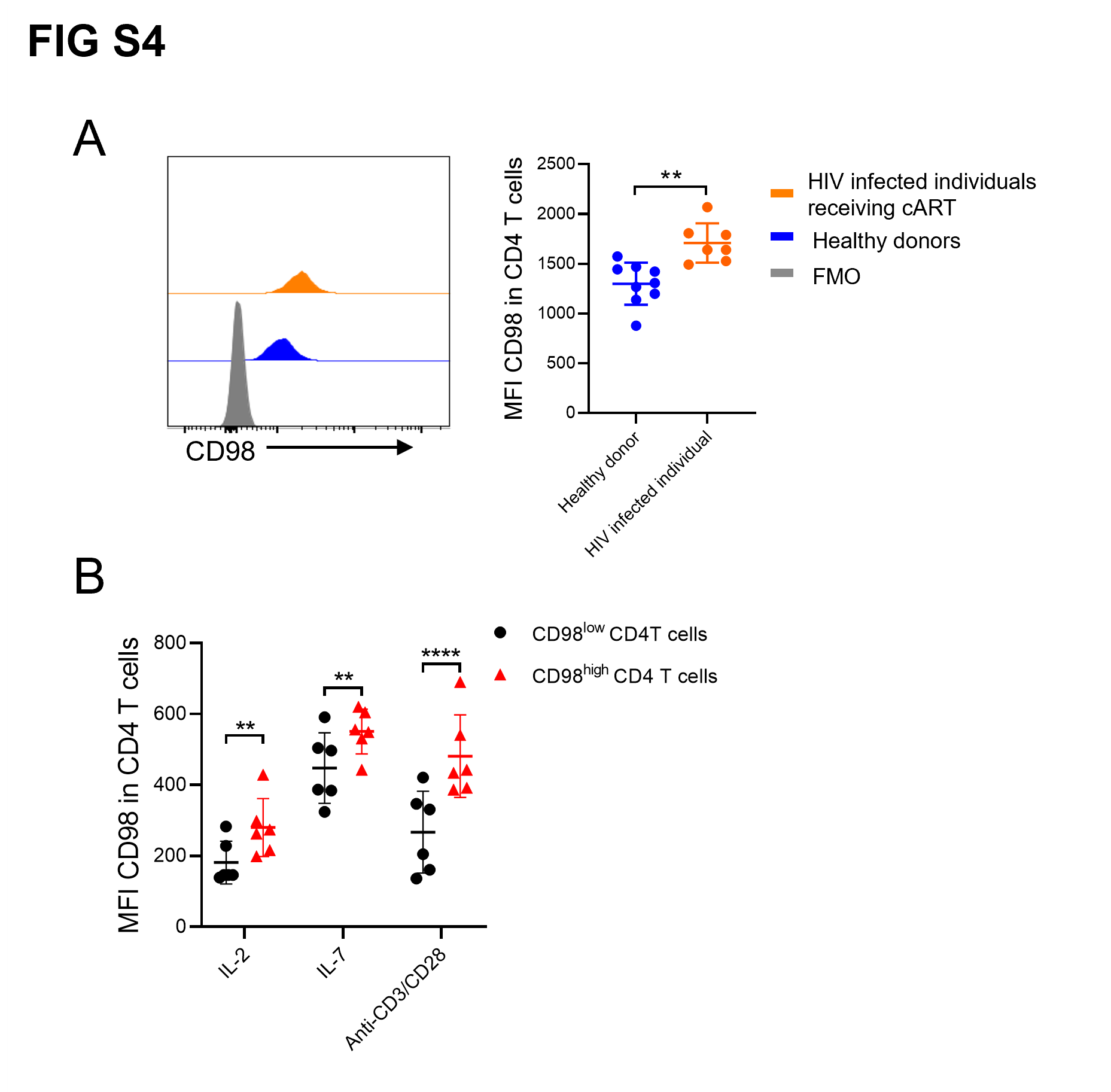

Supplement: FIG S4 [file mbio.02496-22-s0004.tif]

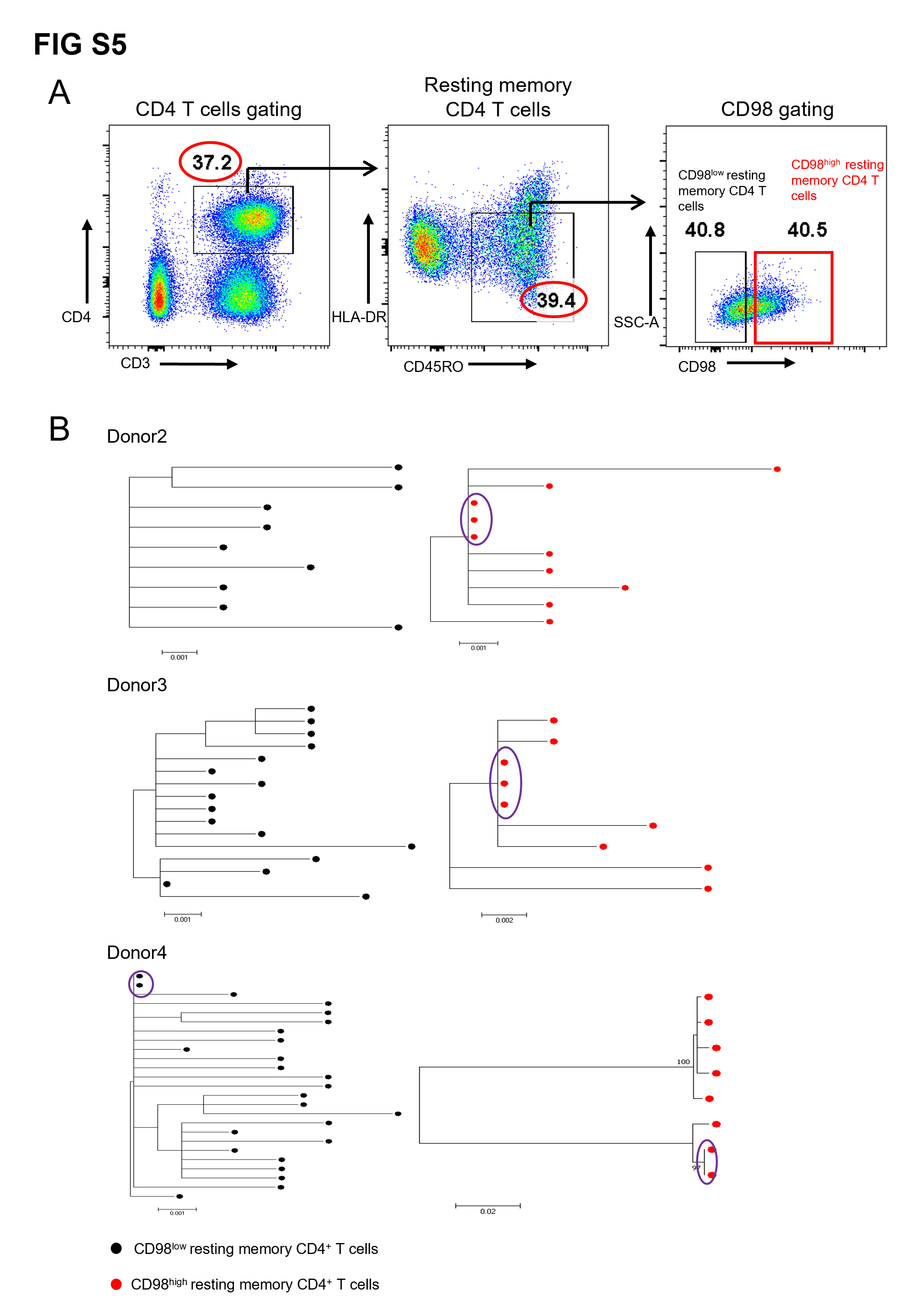

Supplement: FIG S5 [file mbio.02496-22-s0005.tif]

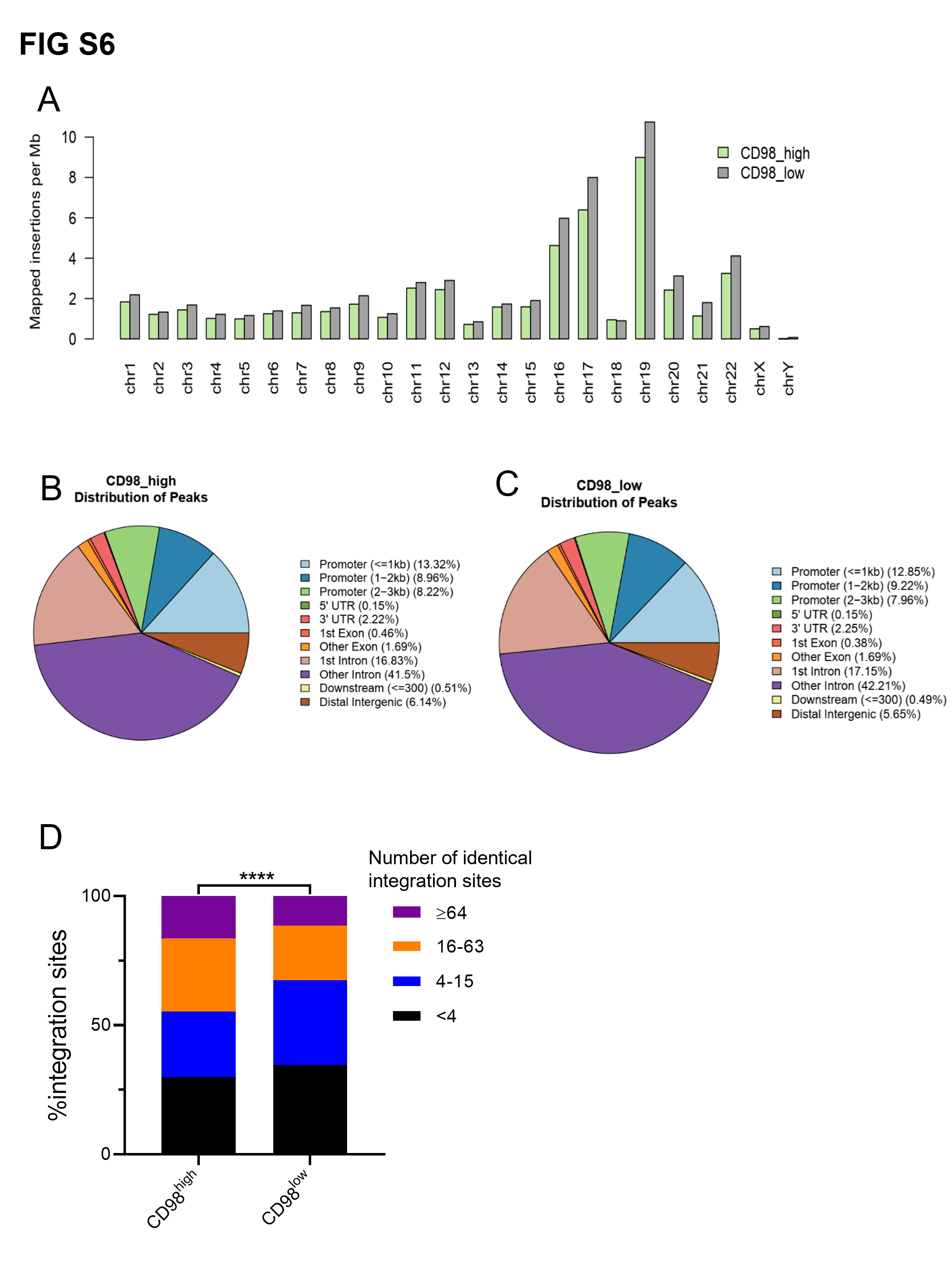

Supplement: FIG S6 [file mbio.02496-22-s0006.tif]
