# Supplementary material for: Identification of CD98 as a Novel Biomarker for HIV-1 Permissiveness and Latent Infection
Source: mBio. 2022 Oct 10;13(6):e02496-22. doi: 10.1128/mbio.02496-22 (PMC9765422; doi:10.1128/mbio.02496-22)
Supplement: TABLE S1 [file mbio.02496-22-s0007.docx]

| **TABLE S1 Upregulated plasma membrane** **proteins on HIV latently infected cells** | | | | |
| --- | --- | --- | --- | --- |
| **Accession** | **Identifier ID** | **Significance** | **Group Profile (Ratio)** | **Description** |
| HINT1 | P49773 | 122.06 | 199.56:183.63:1.00 | Histidine triad nucleotide-binding protein 1 OS=Homo sapiens GN=HINT1 PE=1 SV=2 |
| PAK2 | Q13177 | 105.97 | 20.45:12.23:1.00 | Serine/threonine-protein kinase PAK 2 OS=Homo sapiens GN=PAK2 PE=1 SV=3 |
| CDC42 | P60953 | 99.11 | 1.00:1.46:0 | Cell division control protein 42 homolog OS=Homo sapiens GN=CDC42 PE=1 SV=2 |
| ESYT2 | A0FGR8 | 94.64 | 18.91:1.99:1.00 | Extended synaptotagmin-2 OS=Homo sapiens GN=ESYT2 PE=1 SV=1 |
| 1433F | Q04917 | 93.17 | 20.38:16.90:1.00 | 14-3-3 protein eta OS=Homo sapiens GN=YWHAH PE=1 SV=4 |
| PDS5A | Q29RF7 | 91.65 | 34.84:29.51:1.00 | Sister chromatid cohesion protein PDS5 homolog A OS=Homo sapiens GN=PDS5A PE=1 SV=1 |
| KAP0 | P10644 | 88 | 10.76:8.30:1.00 | cAMP-dependent protein kinase type I-alpha regulatory subunit OS=Homo sapiens GN=PRKAR1A PE=1 SV=1 |
| IQGA2 | Q13576 | 81.45 | 1.00:2.65:0 | Ras GTPase-activating-like protein IQGAP2 OS=Homo sapiens GN=IQGAP2 PE=1 SV=4 |
| FERM2 | Q96AC1-2 | 79.95 | 1.00:1.04:0 | Isoform 2 of Fermitin family homolog 2 OS=Homo sapiens GN=FERMT2 |
| ATAD1 | Q8NBU5 | 73.73 | 27.31:31.62:1.00 | ATPase family AAA domain-containing protein 1 OS=Homo sapiens GN=ATAD1 PE=1 SV=1 |
| PANX1 | Q96RD7 | 73.12 | 1.00:1.38:0 | Pannexin-1 OS=Homo sapiens GN=PANX1 PE=1 SV=4 |
| OSBL2 | Q9H1P3-2 | 73.09 | 1.00:1.12:0 | Isoform 2 of Oxysterol-binding protein-related protein 2 OS=Homo sapiens GN=OSBPL2 |
| VAV | P15498-2 | 72.79 | 1.00:1.04:0 | Isoform 2 of Proto-oncogene vav OS=Homo sapiens GN=VAV1 |
| SYMPK | Q92797 | 67.41 | 5.77:3.14:1.00 | Symplekin OS=Homo sapiens GN=SYMPK PE=1 SV=2 |
| CD123 | Q6ZT62 | 57.08 | 11.08:7.58:1.00 | Cell division cycle protein 123 homolog OS=Homo sapiens GN=CDC123 PE=1 SV=1 |
| Q6ZT62 | P36507 | 55.95 | 15.31:2.50:1.00 | SH3 domain-binding protein 1 OS=Homo sapiens GN=SH3BP1 PE=1 SV=1 |
| MP2K2 | P42331-5 | 55.78 | 8.11:6.55:1.00 | Dual specificity mitogen-activated protein kinase kinase 2 OS=Homo sapiens GN=MAP2K2 PE=1 SV=1 |
| RHG25 | P51148 | 55.12 | 9.16:3.84:1.00 | Isoform 5 of Rho GTPase-activating protein 25 OS=Homo sapiens GN=ARHGAP25 |
| RAB5C | E9PJK1 | 53.99 | 5.54:4.47:1.00 | Ras-related protein Rab-5C OS=Homo sapiens GN=RAB5C PE=1 SV=2 |
| E9PJK1 | P07355 | 52.08 | 4.59:4.96:1.00 | CD81 antigen OS=Homo sapiens GN=CD81 PE=1 SV=1 |
| ANXA2 | Q99653 | 51.81 | 4.32:4.95:1.00 | Annexin A2 OS=Homo sapiens GN=ANXA2 PE=1 SV=2 |
| CHP1 | Q8NFF5-3 | 50.27 | 5.21:1.82:1.00 | Calcineurin B homologous protein 1 OS=Homo sapiens GN=CHP1 PE=1 SV=3 |
| FAD1 | Q96P48-3 | 45.22 | 4.93:4.45:1.00 | Isoform 3 of FAD synthase OS=Homo sapiens GN=FLAD1 |
| ARAP1 | P02649 | 44.56 | 4.84:4.46:1.00 | Isoform 3 of Arf-GAP with Rho-GAP domain ANK repeat and PH domain-containing protein 1 OS=Homo sapiens GN=ARAP1 |
| APOE | Q9BRF8 | 43.99 | 9.83:1.78:1.00 | Apolipoprotein E OS=Homo sapiens GN=APOE PE=1 SV=1 |
| CPPED | Q01650 | 41.57 | 4.15:2.73:1.00 | Calcineurin-like phosphoesterase domain-containing protein 1 OS=Homo sapiens GN=CPPED1 PE=1 SV=3 |
| LAT1 | P39656 | 40.36 | 6.36:5.18:1.00 | Large neutral amino acids transporter small subunit 1 OS=Homo sapiens GN=SLC7A5 PE=1 SV=2 |
| OST48 | P08195-4 | 39.56 | 3.26:2.75:1.00 | Dolichyl-diphosphooligosaccharide--protein glycosyltransferase 48 kDa subunit OS=Homo sapiens GN=DDOST PE=1 SV=4 |
| 4F2 | Q9Y3L5 | 35.53 | 2.73:2.77:1.00 | Isoform 4 of 4F2 cell-surface antigen heavy chain OS=Homo sapiens GN=SLC3A2 |
| RAP2C | P09543 | 33.6 | 6.25:4.00:1.00 | Ras-related protein Rap-2c OS=Homo sapiens GN=RAP2C PE=1 SV=1 |
| CN37 | Q9H0F7 | 29.91 | 2.98:1.38:1.00 | 2' 3'-cyclic-nucleotide 3'-phosphodiesterase OS=Homo sapiens GN=CNP PE=1 SV=2 |
| ARL6 | Q92896-2 | 29.09 | 3.45:4.89:1.00 | ADP-ribosylation factor-like protein 6 OS=Homo sapiens GN=ARL6 PE=1 SV=1 |
| GSLG1 | Q13586 | 28.61 | 3.30:7.82:1.00 | Isoform 2 of Golgi apparatus protein 1 OS=Homo sapiens GN=GLG1 |
| STIM1 | Q8IUI8-2 | 27.21 | 2.72:2.54:1.00 | Stromal interaction molecule 1 OS=Homo sapiens GN=STIM1 PE=1 SV=3 |
| CRLF3 | P55160-2 | 25.6 | 4.65:2.42:1.00 | Isoform 2 of Cytokine receptor-like factor 3 OS=Homo sapiens GN=CRLF3 |
| NCKPL | Q6ZNJ1-2 | 25.47 | 2.76:3.16:1.00 | Isoform 2 of Nck-associated protein 1-like OS=Homo sapiens GN=NCKAP1L |
| NBEL2 | Q15262 | 23.31 | 3.21:2.92:1.00 | Isoform 2 of Neurobeachin-like protein 2 OS=Homo sapiens GN=NBEAL2 |
| PTPRK | Q9UJU6-2 | 22.61 | 11.49:5.54:1.00 | Receptor-type tyrosine-protein phosphatase kappa OS=Homo sapiens GN=PTPRK PE=1 SV=2 |
| DBNL | H7C0I2 | 22.58 | 2.49:1.60:1.00 | Isoform 2 of Drebrin-like protein OS=Homo sapiens GN=DBNL |
| H7C0I2 | P61225 | 21.82 | 4.34:5.63:1.00 | T-cell surface glycoprotein CD1b (Fragment) OS=Homo sapiens GN=CD1B PE=4 SV=1 |
| RAP2B | P14550 | 19.6 | 2.83:2.33:1.00 | Ras-related protein Rap-2b OS=Homo sapiens GN=RAP2B PE=1 SV=1 |
| AK1A1 | Q9Y4H4 | 18.76 | 3.71:4.06:1.00 | Alcohol dehydrogenase [NADP (+)] OS=Homo sapiens GN=AKR1A1 PE=1 SV=3 |
| GPSM3 | P20936-2 | 17.26 | 7.07:4.51:1.00 | G-protein-signaling modulator 3 OS=Homo sapiens GN=GPSM3 PE=1 SV=1 |
| RASA1 | Q8N2K0-2 | 17.08 | 2.42:1.03:1.00 | Isoform 2 of Ras GTPase-activating protein 1 OS=Homo sapiens GN=RASA1 |
| ABD12 | P49755 | 16.96 | 2.65:3.60:1.00 | Isoform 2 of Monoacylglycerol lipase ABHD12 OS=Homo sapiens GN=ABHD12 |
| TMEDA | O00193 | 16.75 | 2.83:2.64:1.00 | Transmembrane emp24 domain-containing protein 10 OS=Homo sapiens GN=TMED10 PE=1 SV=2 |
| SMAP | P16615 | 16.38 | 2.16:1.24:1.00 | Small acidic protein OS=Homo sapiens GN=SMAP PE=1 SV=1 |
| AT2A2 | Q04760-2 | 15.73 | 2.10:1.94:1.00 | Sarcoplasmic/endoplasmic reticulum calcium ATPase 2 OS=Homo sapiens GN=ATP2A2 PE=1 SV=1 |
| LGUL | Q9BWU0 | 15.17 | 1.81:1.96:1.00 | Isoform 2 of Lactoylglutathione lyase OS=Homo sapiens GN=GLO1 |
| NADAP | Q04759 | 13.89 | 1.97:1.82:1.00 | Kanadaptin OS=Homo sapiens GN=SLC4A1AP PE=1 SV=1 |
| KPCT | P49903 | 13.88 | 2.10:1.29:1.00 | Protein kinase C theta type OS=Homo sapiens GN=PRKCQ PE=1 SV=3 |
| SPS1 | Q9UL25 | 13.78 | 2.04:2.91:1.00 | Selenide water dikinase 1 OS=Homo sapiens GN=SEPHS1 PE=1 SV=2 |
| RAB21 | P50552 | 13.67 | 1.93:2.23:1.00 | Ras-related protein Rab-21 OS=Homo sapiens GN=RAB21 PE=1 SV=3 |
| VASP | O43813 | 13.29 | 3.44:1.94:1.00 | Vasodilator-stimulated phosphoprotein OS=Homo sapiens GN=VASP PE=1 SV=3 |
| LANC1 | P00813 | 13.24 | 1.93:1.40:1.00 | LanC-like protein 1 OS=Homo sapiens GN=LANCL1 PE=1 SV=1 |
| ADA | Q5T4S7-2 | 13.09 | 1.77:1.55:1.00 | Adenosine deaminase OS=Homo sapiens GN=ADA PE=1 SV=3 |
| UBR4 | P06744-2 | 12.95 | 1.94:1.77:1.00 | Isoform 2 of E3 ubiquitin-protein ligase UBR4 OS=Homo sapiens GN=UBR4 |
| G6PI | Q9Y295 | 12.11 | 1.68:2.39:1.00 | Isoform 2 of Glucose-6-phosphate isomerase OS=Homo sapiens GN=GPI |
| DRG1 | O15121 | 11.89 | 1.81:1.47:1.00 | Developmentally-regulated GTP-binding protein 1 OS=Homo sapiens GN=DRG1 PE=1 SV=1 |
| DEGS1 | Q8WXH0 | 10.87 | 3.48:2.03:1.00 | Sphingolipid delta (4)-desaturase DES1 OS=Homo sapiens GN=DEGS1 PE=1 SV=1 |
| SYNE2 | P10253 | 10.56 | 1.85:1.80:1.00 | Nesprin-2 OS=Homo sapiens GN=SYNE2 PE=1 SV=3 |
| LYAG | Q12851-2 | 10.45 | 2.10:1.50:1.00 | Lysosomal alpha-glucosidase OS=Homo sapiens GN=GAA PE=1 SV=4 |
| M4K2 | P46934-4 | 10.19 | 2.42:2.25:1.00 | Isoform 2 of Mitogen-activated protein kinase kinase kinase kinase 2 OS=Homo sapiens GN=MAP4K2 |
| NEDD4 | Q96CW1-2 | 9.79 | 5.31:2.96:1.00 | Isoform 4 of E3 ubiquitin-protein ligase NEDD4 OS=Homo sapiens GN=NEDD4 |
| AP2M1 | P18085 | 9.61 | 1.71:1.13:1.00 | Isoform 2 of AP-2 complex subunit mu OS=Homo sapiens GN=AP2M1 |
| ARF4 | Q96EP0 | 9.35 | 1.64:1.05:1.00 | ADP-ribosylation factor 4 OS=Homo sapiens GN=ARF4 PE=1 SV=3 |
| RNF31 | Q14160 | 9.35 | 1.00:2.36:0 | E3 ubiquitin-protein ligase RNF31 OS=Homo sapiens GN=RNF31 PE=1 SV=1 |
| SCRIB | P26038 | 9.28 | 5.13:5.20:1.00 | Protein scribble homolog OS=Homo sapiens GN=SCRIB PE=1 SV=4 |
| MOES | P50570 | 8.89 | 1.64:2.02:1.00 | Moesin OS=Homo sapiens GN=MSN PE=1 SV=3 |
| DYN2 | P14735 | 8.67 | 1.00:1.54:0 | Dynamin-2 OS=Homo sapiens GN=DNM2 PE=1 SV=2 |
| IDE | C9J6F3 | 8.59 | 1.00:1.16:0 | Insulin-degrading enzyme OS=Homo sapiens GN=IDE PE=1 SV=4 |
| C9J6F3 | P55011-3 | 8.51 | 1.15:3.49:1.00 | Programmed cell death protein 10 OS=Homo sapiens GN=PDCD10 PE=1 SV=1 |
| S12A2 | Q96AA3 | 8.37 | 1.00:2.50:0 | Isoform 2 of Solute carrier family 12member 2 OS=Homo sapiens GN=SLC12A2 |
| RFT1 | Q92859-2 | 8.29 | 1.00:1.28:0 | Protein RFT1 homolog OS=Homo sapiens GN=RFT1 PE=1 SV=1 |
| NEO1 | Q92851-2 | 8.18 | 1.00:3.91:0 | Isoform 2 of Neogenin OS=Homo sapiens GN=NEO1 |
| CASPA | P42785 | 8.17 | 1.89:1.42:1.00 | Isoform B of Caspase-10 OS=Homo sapiens GN=CASP10 |
| PCP | Q9BXS5-2 | 7.76 | 1.58:1.53:1.00 | Lysosomal Pro-X carboxypeptidase OS=Homo sapiens GN=PRCP PE=1 SV=1 |
| AP1M1 | Q15628 | 7.57 | 1.61:2.15:1.00 | Isoform 2 of AP-1 complex subunit mu-1 OS=Homo sapiens GN=AP1M1 |
| TRADD | Q10589 | 7.5 | 2.76:1.02:1.00 | Tumor necrosis factor receptor type 1-associated DEATH domain protein OS=Homo sapiens GN=TRADD PE=1 SV=2 |
| BST2 | Q9H0H5 | 6.84 | 2.65:1.01:1.00 | Bone marrow stromal antigen 2 OS=Homo sapiens GN=BST2 PE=1 SV=1 |
| RGAP1 | Q15125 | 6.59 | 3.99:1.14:1.00 | Rac GTPase-activating protein 1 OS=Homo sapiens GN=RACGAP1 PE=1 SV=1 |
| EBP | Q86V85 | 6.4 | 1.80:1.29:1.00 | 3-beta-hydroxysteroid-Delta (8) Delta (7)-isomerase OS=Homo sapiens GN=EBP PE=1 SV=3 |
| GP180 | O75116 | 6.11 | 1.00:1.06:0 | Integral membrane protein GPR180 OS=Homo sapiens GN=GPR180 PE=2 SV=1 |
| ROCK2 | Q9Y487 | 6.1 | 1.00:1.53:0 | Rho-associated protein kinase 2 OS=Homo sapiens GN=ROCK2 PE=1 SV=4 |
| VPP2 | P14317 | 6.09 | 1.00:1.94:0 | V-type proton ATPase 116 kDa subunit a isoform 2 OS=Homo sapiens GN=ATP6V0A2 PE=1 SV=2 |
| HCLS1 | Q96JI7-2 | 6.07 | 1.44:1.01:1.00 | Hematopoietic lineage cell-specific protein OS=Homo sapiens GN=HCLS1 PE=1 SV=3 |
| SPTCS | P11233 | 6.04 | 1.00:3.49:0 | Isoform 2 of Spatacsin OS=Homo sapiens GN=SPG11 |
| RALA | P17612-2 | 6.02 | 2.26:1.86:1.00 | Ras-related protein Ral-A OS=Homo sapiens GN=RALA PE=1 SV=1 |
| KAPCA | Q0VDF9 | 5.8 | 1.50:2.12:1.00 | Isoform 2 of cAMP-dependent protein kinase catalytic subunit alpha OS=Homo sapiens GN=PRKACA |
| HSP7E | Q9UL26 | 5.7 | 2.24:2.06:1.00 | Heat shock 70 kDa protein 14 OS=Homo sapiens GN=HSPA14 PE=1 SV=1 |
| RB22A | Q9UK76 | 5.65 | 1.75:1.17:1.00 | Ras-related protein Rab-22A OS=Homo sapiens GN=RAB22A PE=1 SV=2 |
| HN1 | Q96L92-3 | 5.64 | 1.58:1.15:1.00 | Hematological and neurological expressed 1 protein OS=Homo sapiens GN=HN1 PE=1 SV=3 |
| SNX27 | Q9H4M9 | 5.62 | 1.50:1.08:1.00 | Isoform 2 of Sorting nexin-27 OS=Homo sapiens GN=SNX27 |
| EHD1 | O43665-2 | 5.44 | 1.69:1.39:1.00 | EH domain-containing protein 1 OS=Homo sapiens GN=EHD1 PE=1 SV=2 |
| RGS10 | O76003 | 5.42 | 2.00:2.63:1.00 | Isoform 2 of Regulator of G-protein signaling 10 OS=Homo sapiens GN=RGS10 |
| GLRX3 | P54920 | 5.26 | 2.11:2.37:1.00 | Glutaredoxin-3 OS=Homo sapiens GN=GLRX3 PE=1 SV=2 |
| SNAA | Q13642-5 | 5.06 | 1.72:1.97:1.00 | Alpha-soluble NSF attachment protein OS=Homo sapiens GN=NAPA PE=1 SV=3 |
| FHL1 | P62140 | 4.73 | 1.48:1.49:1.00 | Isoform 5 of Four and a half LIM domains protein 1 OS=Homo sapiens GN=FHL1 |
| PP1B | Q16512 | 4.58 | 1.38:1.19:1.00 | Serine/threonine-protein phosphatase PP1-beta catalytic subunit OS=Homo sapiens GN=PPP1CB PE=1 SV=3 |
| PKN1 | Q14008-2 | 4.44 | 1.57:1.34:1.00 | Serine/threonine-protein kinase N1 OS=Homo sapiens GN=PKN1 PE=1 SV=2 |
| CKAP5 | P27449 | 4.34 | 1.36:1.54:1.00 | Isoform 2 of Cytoskeleton-associated protein 5 OS=Homo sapiens GN=CKAP5 |
| VATL | O75695 | 4.1 | 1.56:1.64:1.00 | V-type proton ATPase 16 kDa proteolipid subunit OS=Homo sapiens GN=ATP6V0C PE=1 SV=1 |
| XRP2 | P07384 | 4 | 1.36:2.13:1.00 | Protein XRP2 OS=Homo sapiens GN=RP2 PE=1 SV=4 |
| CAN1 | Q9H0U3 | 3.56 | 1.28:1.46:1.00 | Calpain-1 catalytic subunit OS=Homo sapiens GN=CAPN1 PE=1 SV=1 |
| MAGT1 | P61106 | 3.47 | 1.28:1.02:1.00 | Magnesium transporter protein 1 OS=Homo sapiens GN=MAGT1 PE=1 SV=1 |
| RAB14 | P53985 | 3.24 | 1.38:1.60:1.00 | Ras-related protein Rab-14 OS=Homo sapiens GN=RAB14 PE=1 SV=4 |
| MOT1 | Q9NPQ8-2 | 2.89 | 1.61:1.04:1.00 | Monocarboxylate transporter 1 OS=Homo sapiens GN=SLC16A1 PE=1 SV=3 |
| RIC8A | Q14108 | 2.85 | 1.74:1.44:1.00 | Isoform 2 of Synembryn-A OS=Homo sapiens GN=RIC8A |
| SCRB2 | Q5T9A4 | 2.84 | 1.61:1.90:1.00 | Lysosome membrane protein 2 OS=Homo sapiens GN=SCARB2 PE=1 SV=2 |
| ATD3B | Q9HB21-2 | 2.71 | 3.00:2.83:1.00 | ATPase family AAA domain-containing protein 3B OS=Homo sapiens GN=ATAD3B PE=1 SV=1 |
| PKHA1 | Q9ULC3 | 2.71 | 1.57:1.78:1.00 | Isoform 2 of Pleckstrin homology domain-containing family A member 1 OS=Homo sapiens GN=PLEKHA1 |
| RAB23 | Q8WU76-2 | 2.65 | 1.27:1.37:1.00 | Ras-related protein Rab-23 OS=Homo sapiens GN=RAB23 PE=1 SV=1 |
| SCFD2 | Q13618 | 2.53 | 1.35:2.46:1.00 | Isoform 2 of Sec1 family domain-containing protein 2 OS=Homo sapiens GN=SCFD2 |
| CUL3 | G3V0E5 | 2.48 | 1.10:2.08:1.00 | Cullin-3 OS=Homo sapiens GN=CUL3 PE=1 SV=2 |
| G3V0E5 | Q7KYR7-6 | 2.37 | 1.25:1.42:1.00 | Transferrin receptor (P90 CD71) isoform CRA_c OS=Homo sapiens GN=TFRC PE=1 SV=1 |
| BT2A1 | Q01469 | 2.31 | 1.26:2.32:1.00 | Isoform 6 of Butyrophilin subfamily 2member A1 OS=Homo sapiens GN=BTN2A1 |
| FABP5 | Q00535 | 2.02 | 1.02:1.40:1.00 | Fatty acid-binding protein epidermal OS=Homo sapiens GN=FABP5 PE=1 SV=3 |
| CDK5 | Q96QD8 | 1.77 | 1.22:1.55:1.00 | Cyclin-dependent kinase 5 OS=Homo sapiens GN=CDK5 PE=1 SV=3 |
| S38A2 | Q9Y6M7-6 | 1.71 | 1.09:1.50:1.00 | Sodium-coupled neutral amino acid transporter 2 OS=Homo sapiens GN=SLC38A2 PE=1 SV=2 |
| S4A7 | P15531 | 1.49 | 1.27:1.64:1.00 | Isoform 6 of Sodium bicarbonate cotransporter 3 OS=Homo sapiens GN=SLC4A7 |
| NDKA | Q9Y6X5 | 1.36 | 1.12:1.03:1.00 | Nucleoside diphosphate kinaseA OS=Homo sapiens GN=NME1 PE=1 SV=1 |
| ENPP4 | Q9H813 | 1.28 | 1.40:1.35:1.00 | Bis(5'-adenosyl)-triphosphatase ENPP4 OS=Homo sapiens GN=ENPP4 PE=1 SV=3 |
| TM206 | Q13303-4 | 1.15 | 1.02:1.40:1.00 | Transmembrane protein 206 OS=Homo sapiens GN=TMEM206 PE=1 SV=1 |
| KCAB2 | Q9UBC2 | 0.83 | 1.09:1.26:1.00 | Isoform 4 of Voltage-gated potassium channel subunit beta-2 OS=Homo sapiens GN=KCNAB2 |
| EP15R | Q96JM3 | 0.67 | 1.07:1.05:1.00 | Epidermal growth factor receptor substrate 15-like 1 OS=Homo sapiens GN=EPS15L1 PE=1 SV=1 |
| CHAP1 | O75794 | 0.59 | 1.05:1.20:1.00 | Chromosome alignment-maintaining phosphoprotein 1 OS=Homo sapiens GN=CHAMP1 PE=1 SV=2 |
