# Supplementary material for: Identification of CD98 as a Novel Biomarker for HIV-1 Permissiveness and Latent Infection
Source: mBio. 2022 Oct 10;13(6):e02496-22. doi: 10.1128/mbio.02496-22 (PMC9765422; doi:10.1128/mbio.02496-22)
Supplement: TABLE S2 [file mbio.02496-22-s0008.docx]

| **TABLE S2 Antibodies for flow cytometry analysis** | | | | | |
| --- | --- | --- | --- | --- | --- |
| Antigen | Specificity | Format | Clone | Brand | Cat |
| CD98 | Human | PE | UM7F8 | BD Pharmingen™ | 556077 |
| CD98 | Human | FITC | MEM-108 | Biolegend | 315603 |
| CD3 | Human | FITC | UCHT1 | Biolegend | 300406 |
| CD3 | Human | PE | UCHT1 | BD Pharmingen™ | 555333 |
| CD4 | Human | APC/Cyanine7 | OKT4 | Biolegend | 317450 |
| CD45RO | Human | APC | UCHL1 | Biolegend | 304210 |
| CD45RO | Human | Alexa Fluor® 700 | UCHL1 | Biolegend | 304218 |
| CD27 | Human | Pacific Blue | M-T271 | BD Horizon™ | 560448 |
| CCR7 | Human | APC | 150503 | BD Pharmingen™ | 560816 |
| HLA DR | Human | Alexa Fluor® 700 | LN3 | eBioscience | 56-9956-42 |
| CCR6 | Human | Pacific Blue | 11A9 | BD Horizon™ | 565925 |
| CCR4 | Human | PE/Cyanine7 | 1G1 | BD Pharmingen™ | 557864 |
| CXCR3 | Human | Percp-cy5.5 | G025H7 | Biolegend | 353713 |
| CXCR5 | Human | BV421 | RF8B2 | Biolegend | 562747 |
| CXCR4 | Human | PE/Cyanine7 | 12G5 | Biolegend | 306513 |
| CCR5 | Human | Alexa Fluor® 700 | J418F1 | Biolegend | 359115 |
| PD-1 | Human | PE/Cyanine7 | A17188B | Biolegend | 621616 |
| TIGIT | Human | Percp-cy5.5 | A15153G | Biolegend | 372718 |
| CD81 | Human | PE | 5A6 | Biolegend | 349505 |
| CD71 | Human | APC | CY1G4 | Biolegend | 334107 |
| CD127 | Human | BV421 | A019D5 | Biolegend | 351309 |
| Ki67 | Human | PE/Cyanine7 | B56 | BD Pharmingen™ | 561283 |
| CD38 | Human | PE/Cyanine7 | HB-7 | Biolegend | 356607 |
| HIV p24 | gag | AF647 |  | Santa Cruz Biotechnology | sc-69728 AF647 |
| Fixable Viability Dye |  | eFluor™ 780 |  | eBioscience | 65-0865-14 |
| CFSE Cell Division Tracker Kit |  |  |  | Biolegend | 423801 |
