# Supplementary material for: Identification of CD98 as a Novel Biomarker for HIV-1 Permissiveness and Latent Infection
Source: mBio. 2022 Oct 10;13(6):e02496-22. doi: 10.1128/mbio.02496-22 (PMC9765422; doi:10.1128/mbio.02496-22)
Supplement: TABLE S3 [file mbio.02496-22-s0009.docx]

**TABLE S2 Gene specific primer sequences**

| **Gene Name** | **Primer Sequence (5'-3')** |
| --- | --- |
| BST2 | Forward, CACACTGTGATGGCCCTAATG |
|  | Reverse, GTCCGCGATTCTCACGCTT |
| CD123 | Forward, ACGAAGGAAGATCCAAACCCA |
|  | Reverse, GCATAGAATAGTCGGCGTCTTTA |
| CD1b | Forward, ATAGCAGGCTGTGAGCTACAT |
|  | Reverse, ACTCAGGAAATCCAATCCTCCTA |
| CD36 | Forward, GGCTGTGACCGGAACTGTG |
|  | Reverse, AGGTCTCCAACTGGCATTAGAA |
| CD81 | Forward, TTCCACGAGACGCTTGACTG |
|  | Reverse, CCCGAGGGACACAAATTGTTC |
| SLC3A2 | Forward, CCAACTACCGGGGTGAGAAC |
|  | Reverse, AGGTCGGAGGAGTTAGTCCC |
| TFR1 | Forward, GGCTACTTGGGCTATTGTAAAGG |
|  | Reverse, CAGTTTCTCCGACAACTTTCTCT |
| β-actin | Forward, GCATGGAGTCCTGTGGCA |
|  | Reverse, CAGGAGGAGCAATGATCTTGA |
| RORC | Forward, CTGGGCATGTCCCGAGATG |
|  | Reverse, GAGGGGTCTTGACCACTGG |
| BCL6 | Forward, ACACATCTCGGCTCAATTTGC |
|  | Reverse, AGTGTCCACAACATGCTCCAT |
| MKI67 | Forward, AGAAGAAGTGGTGCTTCGGAA |
|  | Reverse, AGTTTGCGTGGCCTGTACTAA |
| BIRC5 | Forward, CAAGGACCACCGCATCTCTA |
|  | Reverse, TGTTCCTCTATGGGGTCGTCA |
| IL17 | Forward, TCCCACGAAATCCAGGATGC |
|  | Reverse, GGATGTTCAGGTTGACCATCAC |
| IL22 | Forward, CTCTTGGCCCTCTTGGTACAG |
|  | Reverse, CGCTCACTCATACTGACTCCG |
| TNFRSF4 | Forward, GCAATAGCTCGGACGCAATCT |
|  | Reverse, GAGGGTCCCTGTGAGGTTCT |
| SLC7A5 | Forward, GGAAGGGTGATGTGTCCAATC |
|  | Reverse, TAATGCCAGCACAATGTTCCC |
| ITGA1 | Forward, GTGCTTATTGGTTCTCCGTTAGT |
|  | Reverse, CACAAGCCAGAAATCCTCCAT |
| TIGB1 | Forward, CCTACTTCTGCACGATGTGATG |
|  | Reverse, CCTTTGCTACGGTTGGTTACATT |
| KLRB1 | Forward, CCCTTGGAATAACAGTCTAGCTG |
|  | Reverse, TTGTCACGTATCAGGTTCTGTG |
