# Supplementary material for: Identification of CD98 as a Novel Biomarker for HIV-1 Permissiveness and Latent Infection
Source: mBio. 2022 Oct 10;13(6):e02496-22. doi: 10.1128/mbio.02496-22 (PMC9765422; doi:10.1128/mbio.02496-22)
Supplement: TABLE S4 [file mbio.02496-22-s0010.docx]

| **TABLE S4 Sequences of the primers and linkers for integration site analysis** | |
| --- | --- |
| **Primer Name** | **Primer Sequence (5'-3')** |
| HIV-3LTR-outer | TGTGACTCTGGTAACTAGAGATCCCTC |
| HIV-5LTR-outer | TCAGGGAAGTAGCCTTGTGTGTGGT |
| HIV-3LTRnest-Inner | AATGATACGGCGACCACCGAGATCTACACTCTTTCCCTACACGACGCTCTTCCGATCTCCCTTTTAGTCAGTGTGGAAAATC |
| HIV-5LTRnest-Inner | AATGATACGGCGACCACCGAGATCTACACTCTTTCCCTACACGACGCTCTTCCGATCTTGTCTTTTTTGGGACCAAATTAGC |
| Linker-outer | AGTTCAGACGTGTGCTCTTC |
| PE2_Linkernest-inner | CAAGCAGAAGACGGCATACGAGATCGTAGATCGTGACTGGAGTTCAGACGTGTGCTCTTCCGATC |
| A partially double stranded linker-1 | AGTTCAGACGTGTGCTCTTCCGATCTATTACTCGNNNNNNNNNNTAGTGCTCCGCTTAGAGGACT |
| A partially double stranded linker-2 | 5'Phos-GTCCTCTAAGCGGAGCACTA-3'NH2 |
